# *Francisella tularensis* Subtype A.II Genomic Plasticity in Comparison with Subtype A.I

**DOI:** 10.1371/journal.pone.0124906

**Published:** 2015-04-28

**Authors:** Marilynn A. Larson, Ufuk Nalbantoglu, Khalid Sayood, Emily B. Zentz, Amanda M. Bartling, Stephen C. Francesconi, Paul D. Fey, Michael P. Dempsey, Steven H. Hinrichs

**Affiliations:** 1 Department of Pathology and Microbiology, University of Nebraska Medical Center, Omaha, Nebraska, United States of America; 2 Department of Electrical Engineering, University of Nebraska-Lincoln, Lincoln, Nebraska, United States of America; 3 OpGen Inc., Gaithersburg, Maryland, United States of America; 4 Naval Medical Research Center, Fort Detrick, Maryland, United States of America; 5 United States Air Force School of Aerospace Medicine, Wright-Patterson Air Force Base, Ohio, United States of America; University of Louisville, UNITED STATES

## Abstract

Although *Francisella tularensis* is considered a monomorphic intracellular pathogen, molecular genotyping and virulence studies have demonstrated important differences within the *tularensis* subspecies (type A). To evaluate genetic variation within type A strains, sequencing and assembly of a new subtype A.II genome was achieved for comparison to other completed *F*. *tularensis* type A genomes. In contrast with the *F*. *tularensis* A.I strains (SCHU S4, FSC198, NE061598, and TI0902), substantial genomic variation was observed between the newly sequenced *F*. *tularensis* A.II strain (WY-00W4114) and the only other publically available A.II strain (WY96-3418). Genome differences between WY-00W4114 and WY96-3418 included three major chromosomal translocations, 1580 indels, and 286 nucleotide substitutions of which 159 were observed in predicted open reading frames and 127 were located in intergenic regions. The majority of WY-00W4114 nucleotide deletions occurred in intergenic regions, whereas most of the insertions and substitutions occurred in predicted genes. Of the nucleotide substitutions, 48 (30%) were synonymous and 111 (70%) were nonsynonymous. WY-00W4114 and WY96-3418 nucleotide polymorphisms were predominantly G/C to A/T allelic mutations, with WY-00W4114 having more A+T enrichment. In addition, the A.II genomes contained a considerably higher number of intact genes and longer repetitive sequences, including transposon remnants than the A.I genomes. Together these findings support the premise that *F*. *tularensis* A.II may have a fitness advantage compared to the A.I subtype due to the higher abundance of functional genes and repeated chromosomal sequences. A better understanding of the selective forces driving *F*. *tularensis* genetic diversity and plasticity is needed.

## Introduction

The facultative intracellular pathogen *Francisella tularensis* is the etiologic agent of the zoonotic disease tularemia and is considered a Tier 1 select agent by the Centers for Disease Control and Prevention for several reasons: an extremely low infectious dose (10 organisms), prior use as a biological weapon, and the mass casualties and economic impact that may result in the event of a tularemia outbreak, all of which warrant the concern generated by this highly virulent pathogen [1–3]. Although *F*. *tularensis* is most lethal when inhaled, transmission to humans and at least 250 other species can occur via direct contact with contaminated hosts, water, and food (reviewed in [4]). Common reservoirs and vectors for this gram-negative cocco-bacillus in the environment include lagomorphs (primarily rabbits and hares), rodents, and arthropods (primarily ticks, deer flies, and mosquitoes) [3].

Within the *F*. *tularensis* species, there are three widely accepted subspecies that include *F*. *tularensis* subsp. *tularensis* (type A), *F*. *tularensis* subsp. *holarctica* (type B), and *F*. *tularensis* subsp. *mediasiatica* [5]. A fourth subspecies, specifically *F*. *tularensis* subsp. *novicida*, is considerably less virulent than the other subspecies and the classification of this related bacterium remains in contention [6,7]. Differences in genomic architecture and virulence observed for *F*. *tularensis* type A isolates has prompted further subdivision of this subspecies into subtypes A.I and A.II, with the A.I strains having the highest virulence [8–10]. However, the mechanisms responsible for the genotypic and phenotypic differences associated with *F*. *tularensis* A.I and A.II strains are unknown. Infections by *F*. *tularensis* subtype A.I strains have been documented to occur throughout North America, whereas A.II infections have predominately occurred in western United States.

Although the complete and annotated genomic sequence is available for several wild-type *F*. *tularensis* A.I strains (SCHU S4, NE061598, FSC198, and TI0902), only the wild-type A.II strain WY96-3418 (hereafter referred to as WY96) had been sequenced to completion [11–15]. As part of a large sequencing project, we previously detected a wild-type *F*. *tularensis* subsp. *tularensis* A.II variant using paired-end sequence mapping [16]. This isolate was identified as *F*. *tularensis* WY-00W4114 (hereafter referred to as W4114) and further characterized by pulsed-field gel electrophoresis (PFGE) and differential insertion sequence amplification (DISA) [17]. Even though both the PFGE clustering algorithm and the PCR-based DISA assay classified this isolate as an A.II strain, considerable chromosomal diversity existed in W4114 when compared to other strains within this subtype; in addition, the A.II subpopulation possessed greater diversity in PFGE typing patterns than the A.I clade [17].

The isolation date of all *F*. *tularensis* strains under consideration is important for interpreting the genetic information. *F*. *tularensis* A.I strains SCHU S4 and NE061598 were obtained from an infected human in Ohio (1941) and in Nebraska (1998), respectively, whereas FSC198 was acquired in 1986 from a mite in Slovakia [13,14,18]. The fourth sequenced A.I strain, TI0902, was isolated in 2004 from an infected cat in Virginia [15]. The *F*. *tularensis* A.II strain WY96 was obtained in 1996 from an infected human [12], while W4114 was isolated in 2000 from an infected prairie dog, both of which were originally acquired from the Wyoming public health laboratory.

Based on the literature and our previous findings, we hypothesized that *F*. *tularensis* A.II strains would have greater genetic variation than the A.I strains, revealing molecular mechanisms that correlate with less virulence and that provide a selective advantage in the environment. Therefore, to examine the differences within *F*. *tularensis* type A strains, we sequenced the genome of wild-type AII strain W4114 for comparison to the sequenced A.II strain WY96 and A.I chromosomes. We found that the A.II strains W4114 and WY96 possessed numerous chromosomal polymorphisms and rearrangements, demonstrating genomic plasticity, whereas the A.I chromosome that contained remarkably high genomic conservation. Collectively, this study reveals important genetic differences between the *F*. *tularensis* A.I and A.II subtypes that may contribute to disparities in virulence and fitness.

## Materials and Methods

### Bacterial strains and DNA isolation

*F*. *tularensis* subsp. *tularensis* strain W4114 was transferred to the University of Nebraska Medical Center in Omaha following the requirements of the Select Agent Program as outlined in the Animal and Plant Health Inspection Service/Centers for Disease Control and Prevention (CDC) Form 2, Guidance Document for Request to Transfer Select Agents and Toxins [2]. Manipulation of viable culture material was performed by authorized individuals within a biosafety level 3 laboratory certified for select agent work by the United States Department of Health and Human Services using laboratory biosafety criteria, according to requirements of the national Select Agent Program [19]. All *Francisella* isolates were grown on chocolate agar plates (Remel, Lenexa, Kansas) and incubated at 37°C with 5% CO_2_ for three days before processing.

### Pulsed-field gel electrophoresis

Agarose embedded chromosomal DNA for PFGE was prepared and digested with the restriction endonucleases *Pme*I and *Bam*HI (Fermentas Inc., Glen Burnie, Maryland), as previously described [17]. Cluster analysis was performed using the Dice correlation coefficient and the unweighted pair group mathematical average (UPGMA) clustering algorithm. Restriction fragment length polymorphism analysis was performed using Bionumerics software (Applied Maths, Inc., Austin, Texas).

### Differential insertion sequence amplification

The PCR-based DISA assay was performed using defined primer sets, as previously described [17]. In brief, the unique spatial location of specific insertion sequence (IS) elements and the resulting sizes of the amplified regions provided the ability to identify and distinguish the virulent *F*. *tularensis* subsp. *tularensis* (subtypes A.I and A.II) and subsp. *holarctica* (type B) strains from *F*. *tularensis* subsp. *novicida* and other near neighbors, including *F*. *philomiragia* and *Francisella*-like endosymbionts found in ticks.

### Genome sequencing and assembly

Genomic sequence data for *F*. *tularensis* W4114 was obtained using 454 pyrosequencing at the Naval Medical Research Center with an average read length of 381 bases. At the end of the draft sequence phase, 193,654,006 base pairs (bp) were produced, representing an estimated 108-fold coverage with a Q39 score of 0.03%. *De novo* assembly without using a reference sequence resulted in 82 total contiguous sequences. Primer walk reads using Sanger sequencing were performed as needed to address low quality regions of the draft assembly and to obtain or confirm nucleotide sequence content. After the assembly was completed and verified, the final genome with no gaps was comprised of 1,899,252 bp. The total number of reads used in the final assembly was 25,531. All polymorphisms noted in the newly sequenced W4114 genome in comparison to the reference strain WY96 were validated by at least two independent methods. Further, to verify that these nucleotide differences were not due to errors in the database for WY96, approximately 10% of the polymorphisms in this reference strain were confirmed by PCR amplification of the region of interest and subsequent DNA sequencing.

### Whole genome mapping

High molecular weight genomic DNA from *F*. *tularensis* W4114 was prepared directly from nonviable cell pellets containing approximately 10^7^ cells using the Argus HMW DNA Isolation Kit (OpGen, Inc., Gaithersburg, Maryland). In brief, cells were lysed as recommended by the manufacturer and diluted for direct use. To reduce DNA shearing, wide-bore pipette tips were used and DNA samples were gently mixed without vortexing. DNA was visually inspected for quality and concentration using the Argus QCard, according to the manufacturer’s instructions. The software program Enzyme Chooser (OpGen, Inc.) was used to identify restriction endonuclease cleavage sites in the reference genome that would result in fragments that average 6–12 kilobase pairs (kbp) in size and that would not produce any fragments larger than 80 kbp, if digested with the respective enzyme. Based on the reference strain *F*. *tularensis* WY96, the Argus enzymes *Nco*I and *Nhe*I were chosen and used to restriction map the genome of *F*. *tularensis* W4114 with the Whole Genome Mapping System from OpGen, Inc.

Single genomic DNA molecules were captured onto an Argus surface within a MapCard (OpGen, Inc.) and then digested with the selected Argus restriction enzyme (OpGen, Inc.). The digested DNA was stained with JOJO-1 (Invitrogen, Carlsbad, California) using the Argus MapCard Processor (OpGen, Inc.), and then analyzed by automated fluorescent microscopy using the Argus Whole Genome Mapper (OpGen, Inc.). This software records the size and order of restriction fragments for each DNA molecule. The single molecule restriction map collections were then assembled according to overlapping fragment patterns to produce a consensus Whole Genome Map.

### DNA sequence alignment using MapSolver

DNA sequence data for *F*. *tularensis* W4114 in FASTA formatted files were imported into MapSolver software (OpGen, Inc.) and converted into *in silico* maps using the same restriction enzyme that was used to generate the respective Whole Genome Map. The contiguous DNA sequences and the final assembled genome sequence were then aligned to the Whole Genome Map from *F*. *tularensis* W4114 using the sequence placement function of MapSolver. This dynamic alignment algorithm generates a final alignment score based on the fragment patterns and sizes. In brief, MapSolver finds the optimal alignment of two restriction maps according to a scoring model that incorporates fragment sizing errors, false and missing cuts, and missing small fragments. The number of aligned fragments in an alignment depends on the nearness of the match and the MapSolver alignment settings. By default, the initial minimum is determined by the advanced option of “minimum aligned cuts”, which was set to 4 and corresponds to 3 fragments. The alignment score for every pair of matched fragments can be up to a score of 1 if the fragments perfectly match in size. As the fragment sizes diverge in size, the scoring function awards a lower score, whereas longer alignments between more similar restriction patterns produce a higher score.

### Annotation

For functional annotation, the complete chromosome of Francisella *tularensis* subsp. *tularensis* W4114 was submitted to the National Center for Biotechnology Information (NCBI) Prokaryotic Genomes Automatic Annotation Pipeline (http://www.ncbi.nlm.nih.gov/genomes/static/Pipeline.html). The annotated genes were subsequently confirmed by comparing each open reading frame (ORF) to the respective homolog in WY96 using the standard Smith-Waterman algorithm [20]. Hits attaining higher than 98% identity were considered accurate annotations. The genes predicted by the pipeline that were not annotated in WY96 were considered novel genes. Annotated IS elements that are specific to *F*. ***tularensis*** were obtained from the Universal Protein Resource (UniProt) database (http://www.ebi.ac.uk/uniprot/) and located within the W4114 chromosome using the NCBI Basic Local Alignment Search Tool (BLAST).

### Genome alignments and rearrangements

Locally collinear blocks (LCBs) were determined using the progressiveMauve tool [21]. The parameters for the discovery of LCBs included setting the block search mode to reversals (inversions) plus block interchange mode; the minimum Multiple-Maximal Unique Matches (multi-MUMs) length equal to 21 bp (closest integer to log2, 1898 kbp), where 1,898 kbp is the average genome length); the minimum length of the LCBs equal to 63 bp (3 x minimum multi-MUMs); and the chromosome type as linear. The boundaries of the LCBs were refined by aligning 5 kbp of the flanking regions using BLAST. Boundary changes were determined by an exhaustive search of the change points that gave the maximum alignment score.

### Nucleotide substitution and indel discovery

Nucleotide polymorphisms were discovered between W4114 and WY96 by global alignment of the defined LCBs using the Needleman-Wunsch algorithm with the parameters of 1 mismatch and -2 existence and linear gap costs [22]. All nucleotide polymorphisms in W4114 and 10% of the nucleotide polymorphisms denoted in the NCBI database for WY96 (GenBank accession number CP000608) were each confirmed independently by DNA sequencing. The resulting nucleotide polymorphisms were annotated along with their nucleotide position, intergenic or intragenic location, and predicted effect on the translated amino acid (synonymous versus non-synonymous).

### Identification of variable-number tandem repeats

Tandem repeats within the chromosome of W4114 were identified utilizing the Tandem Repeats Finder algorithm [23]. The number of adjacent tandem repeat motifs and composition differences within the genomes of W4114 and WY96 were reported (see S10 Table).

### Data availability

The complete genome sequence for *F*. *tularensis* subsp. *tularensis* (subtype A.II) strain WY-00W4114 was deposited in GenBank, annotated, and assigned accession number CP009753.

## Results

### Sequencing and whole genome mapping of a novel *F*. *tularensis* A.II strain

Although PFGE typing of *F*. *tularensis* W4114 indicated that this strain belonged to the A.II subtype [16,17], restriction fragment patterns revealed noteworthy differences compared to other A.II strains (S1 Fig). At least five restriction fragments differed between W4114 and two other A.II strains, specifically WY96 and the unsequenced strain WY-06F12590. To examine the apparent genomic plasticity within the *F*. *tularensis* A.II clade in more detail, the chromosome of the unique W4114 strain was sequenced and assembled to completion for subsequent comparison to other fully sequenced type A strains. Computational tools were used for the analyses and the identified polymorphisms were each verified independently. The newly sequenced genome of *F*. *tularensis* A.II strain W4114 was assigned GenBank accession number CP009753.

The nucleotide sequence of *F*. *tularensis* W4114 was acquired by 454 pyrosequencing in which the only other available sequenced A.II strain, specifically WY96 (GenBank accession number CP000608) was used as a reference during the assembly process. Completing the chromosomal sequence for W4114 was problematic due to: (i) the apparent dissimilarity between these two A.II strains, (ii) the inherent limit in length of accurate sequencing reads, and (iii) the high abundance of repeated sequences, particularly the IS elements IS*Ftu1* and IS*Ftu2*. To address these challenges, whole genome mapping (OpGen, Inc) was used to produce a contiguous restriction map of the actual W4114 chromosome for comparison to the newly assembled *in silico* sequence. This methodology facilitated genome closure by identifying problem areas and by validating the final genomic structure.

**Fig 1:**
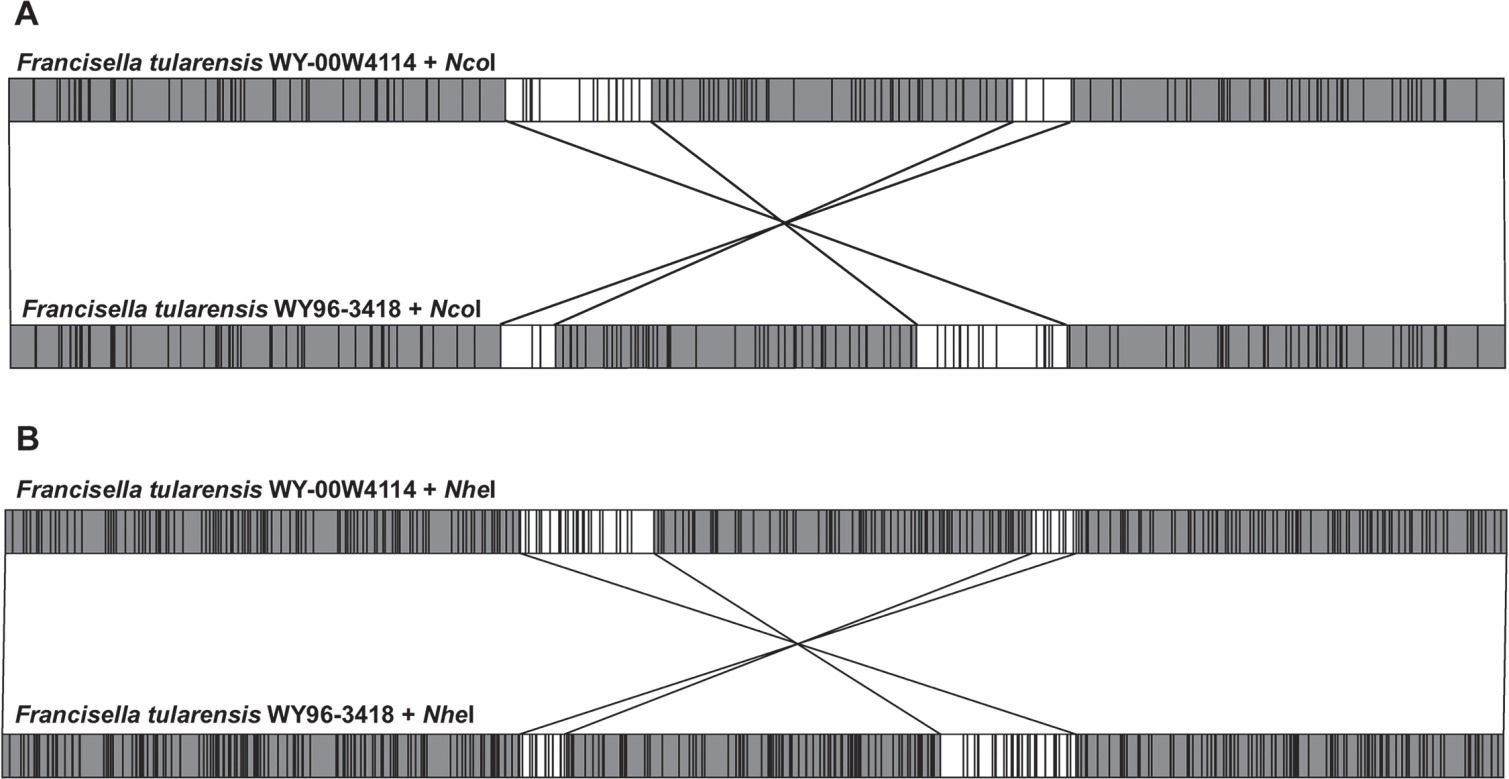
Whole genome mapping of *F*. *tularensis* subtype A.II strains WY-00W4114 and WY96-3418. *Nco*I (A) and *Nhe*I (B) whole genome maps of *F*. *tularensis* WY-00W4114 (top linearized chromosome) compared to the corresponding theoretical *in silico* digestion of *F*. *tularensis* WY96-3418 (GenBank accession number CP000608, bottom linearized chromosome). Vertical lines within the genome maps denote the restriction endonuclease sites for *Nco*I (A) or *Nhe*I (B). Lines connecting the chromosomal restriction maps of WY-00W4114 and WY96-3418 and the adjacent unshaded genomic areas denote translocated regions.

The initial whole genome mapping of *F*. *tularensis* W4114 DNA was performed with *Nco*I (Fig 1A). However, since several chromosomal locations did not have a sufficient number of *Nco*I restriction sites, assembled sequences within these regions could not be confirmed. Therefore, a second whole genome map of W4114 was acquired using *Nhe*I (Fig 1B). When the *Nco*I-digested and *Nhe*I-digested whole genome maps for *F*. *tularensis* W4114 were compared to the respective *in silico* restriction maps of *F*. *tularensis* WY96, several chromosomal rearrangements were apparent (Fig 1).

### Size and topological comparison of *F*. *tularensis* A.II genomes

The *F*. *tularensis* W4114 circular chromosome was 1,899,252 bp in size and 776 bp larger than the WY96 genome (Table 1). Although numerous nucleotide polymorphisms were observed between these two A.II strains, nucleotide insertions predominantly contributed to the size difference noted for the genome of W4114 relative to WY96 (Table 1). The majority of these nucleotide insertions were located within intragenic regions, whereas most of the nucleotide deletions were present within intergenic regions. Further, many nucleotide substitutions were also apparent in both intragenic and intergenic regions when comparing the A.II chromosomes (Table 1).

**Table 1:** Nucleotide polymorphisms in genome of WY-00W4114 compared to WY96-3418 and overall base pair size increase in WY-00W4114.

Nucleotide Polymorphism	Intergenic	Intragenic	Total No.
Insertions	374	804	1178
Deletions	265	137	402
Substitutions	127	159	286
Overall Size Increase	109	667	776

**Fig 2:**
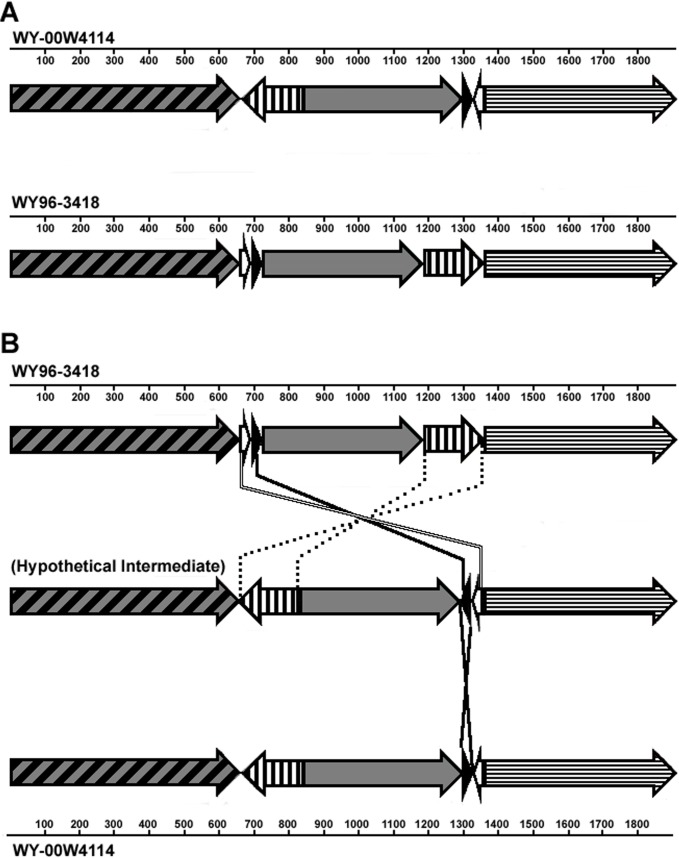
Diagram depicting large rearrangements of locally collinear blocks (LCBs) within *F*. *tularensis* A.II strains. *F*. *tularensis* A.II strains WY-00W4114 and WY96-3418 chromosomal comparison showing related LCBs (A) and potential recombination events with a two-step parsimonious molecular process (B). Each LCB is represented with a different pattern and/or shading. Directionality of the LCBs is depicted with an arrow and is based on the reference strain WY96-3418 (GenBank accession number CP000608). Nucleotide positions are denoted in kilobase pairs by the corresponding genome.

A total of six LCBs were identified in the genome of *F*. *tularensis* W4114 and WY96 (Table 2), with the W4114 chromosome showing the translocation of four LCBs (Fig 2A). Potential recombination events with a two-step parsimonious molecular process were reconstructed and are shown in Fig 2B. Alternatively, an intermediate strain could have given rise to either W4114 or WY96. Inspection of the recombination breakpoints revealed the presence of the two most abundant IS elements, specifically IS*Ftu1* and IS*Ftu2*, on one or both sides of these junctions. Therefore, the chromosomal translocations appeared to be IS element-mediated, as was previously noted when genome comparisons were made between other *F*. *tularensis* strains [13,16,24,25].

**Table 2:** Description and nucleotide position of locally collinear blocks (LCBs) within A.II strains WY-00W4114 and WY96-3418.

LCB	Type	WY-00W4114 Position	WY96-3418 Position
1	Conserved	1–661043	1–660657
2	Inversion	661044–829377	1358861–1190506
3	Conserved	829378–1305560	714449–1190505
4	Rearrangement	1305561–1325296	694742–714448
5	Inversion	1325297–1359385	694741–660658
6	Conserved	1359386–1899270	1358862–1898476

### Characterization of genomic polymorphisms within *F*. *tularensis* A.II strains

The larger chromosomal size for *F*. *tularensis* W4114 relative to WY96 was predominantly due to an increase in A+T content (S1 Table). A direct comparison of the nucleotide composition between these two A.II genomes showed that although the insertion of adenines and thymines was more common in the W4114 chromosome, these two nucleotides were also more often deleted (S2 Table). Guanines and cytosines were also more often inserted than deleted, contributing to the overall size increase observed for the W4114 genome (S1 and S2 Tables). The majority of allelic substitutions in both W4114 and WY96 were transitions in which a cytosine was replaced with a thymine, with the second most frequent change being the exchange of an adenine to a guanine (S3 Table).

Characterization of the genomic indels in *F*. *tularensis* W4114 relative to WY96 showed that a single base pair indel occurred most frequently, and indels longer in length generally occurred only one time (S4 Table). Although the majority of the 44 single base pair indels were within intergenic regions, six were within open reading frames (ORFs) encoding IS*Ftu1* and five others occurred in ORFs annotated to encode proteins (S5 Table). Indels larger than a single base pair occurred in 16 annotated genes, including eight hypothetical proteins, one 5S ribosomal RNA (rRNA) ORF in duplicated region 2 (DR2), and one IS*Ftu1* ORF (S5 and S6 Tables). A 28 bp indel represented the mode with 5 occurrences, all of which were in intergenic regions, and a single 201 bp sequence in a gene encoding a hypothetical protein was the largest indel.

Of the 286 nucleotide substitutions in the *F*. *tularensis* W4114 genome relative to WY96, 159 were observed in predicted intragenic regions and 127 were located in intergenic regions (Table 1 and S7 Table). Forty-eight (30%) of the 159 intragenic substitutions were synonymous and 111 (70%) substitutions were nonsynonymous. Of the 111 nonsynonymous substitutions, only 16 were conservative. The majority of the intragenic nucleotide substitutions were located within IS*Ftu1* ORFs in which 16 of the 56 were synonymous and 40 were nonsynonymous mutations (S7 Table). All 40 nonsynonymous substitutions in the IS*Ftu1* ORFs were missense mutations. The remaining 103 intragenic nucleotide substitutions produced 32 synonymous and 71 nonsynonymous mutations in 91 additional ORFs, including 17 hypothetical proteins (S7 Table). The 32 synonymous mutations occurred in 31 of these ORFs, five of which were predicted to encode a hypothetical protein. Nonsynonymous substitutions occurred in 62 genes and 16 were conservative, including a mutation in the gene encoding the pathogenicity determinant protein PdpC in DR2. In W4114, the nucleotide substitution in *pdpC* resulted in an isoleucine in DR2, and in WY96 DR2 this mutation produced a valine (S7 Table). One nonsynonymous substitution produced a nonsense mutation that resulted in a stop codon within one of the duplicated genes predicted to encode an anhydro-N-acetylmuramic acid kinase in W4114 DR2. Structural RNA genes encoding 16S rRNA in DR2 of W4114 and a serine transfer RNA (tRNA) also contained nucleotide substitutions (S7 Table). Although numerous nucleotide polymorphisms were identified in the *F*. *tularensis* W4114 and WY96 chromosomal comparisons, the effect of these mutations on regulation and/or protein function remains to be determined. Nevertheless, these findings collectively illustrated genomic plasticity within the A.II clade.

### Gene content comparison in *F*. *tularensis* A.II strains

The *F*. *tularensis* W4114 genome contained two additional ORFs relative to the *F*. *tularensis* WY96 chromosome, one encoding a hypothetical protein and the other encoding a quinone-oxidoreductase. In contrast, the WY96 chromosome did not have any ORFs that W4114 did not contain. In both *F*. *tularensis* W4114 and WY96, 46 ORFs were disrupted by an IS element. These interrupted genes were predicted to encode a glycosyl hydrolase, a restriction endonuclease, a kinase, transporters, dehydrogenases, synthases, phosphatases, transferases, aminopeptidases, permeases, methylases, ATPases, and 14 hypothetical proteins (S8 and S9 Tables).

Variable-number tandem repeat (VNTR) markers are often used to study genetic diversity and to distinguish strains of bacterial pathogens (e.g., multiple-locus VNTR analysis or MLVA). A comparison of the VNTR markers within *F*. *tularensis* A.II W4114 and WY96 revealed that a six nucleotide sequence motif was most often repeated with two different 60-mer sequences being the longest VNTR in both strains (S10 Table). However, the repeat copy number and the number of times the VNTR markers were in the A.II genomes differed (S10 Table). For example, in W4114 one of the 60 nucleotide motifs was sequentially repeated twice, but only located at one site in the genome. In comparison, the same VNTR in WY96 was not repeated successively, but was present in the genome at 48 different locations. Another 60 nucleotide VNTR marker was repeated twice in W4114 at a single location in the chromosome, whereas in WY96 this sequence motif was not sequentially repeated, but was present at nine different genomic sites. Additional VNTR differences in the *F*. *tularensis* A.II strains were apparent and are shown in S10 Table.

The strikingly high intraclade abundance of polymorphisms within the *F*. *tularensis* A.II strains W4114 and WY96 was not apparent in the A.I strains [11,13–15]. Therefore, to provide more insight on factors that may contribute to the virulence differences noted between these two *F*. *tularensis* type A subpopulations, an interclade genome comparison was performed.

### *F*. *tularensis* A.I and A.II chromosomal topology and rearrangements

**Fig 3:**
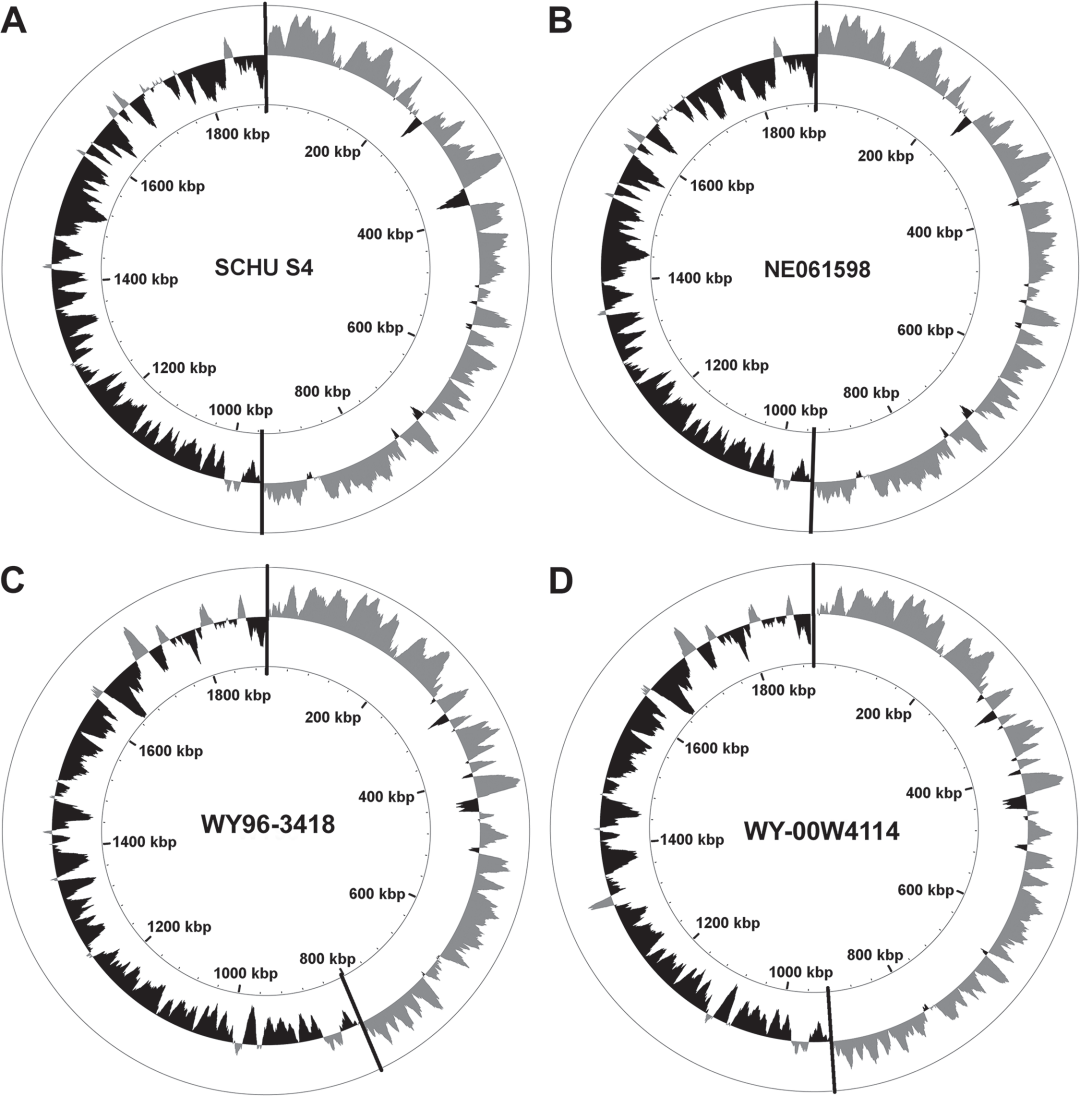
Diagram illustrating GC skew within chromosomal topology map for *F*. *tularensis* A.I and A.II strains. The circular *F*. *tularensis* chromosome of subtype A.I strains are represented by SCHU S4 (A) and NE061598 (B), and WY96-3418 (C) and WY-00W4114 (D) represent the subtype A.II strains. The origin (*ori*) and termination (*ter*) region are denoted by a vertical black line at the top and bottom, respectively, of the corresponding chromosomal map. GC skew + (gray) and GC skew—(black) is shown in the outermost circle for each genome and the kilobase pair position is indicated in the innermost circle.

GC skew in the chromosome of *F*. *tularensis* subtype A.I strains SCHU S4 and NE610598 and subtype A.II strains W4114 and WY96 was similar within members of the same subtype, but differed between these two clades (Fig 3). However, the termination (*ter*) region within the A.II strains differed considerably due to an apparent genomic rearrangement. The spatial location of the *ter* region in W4114, which is depicted by the change in GC skew, correlated more with the A.I strains than with the A.II strain WY96.

**Fig 4:**
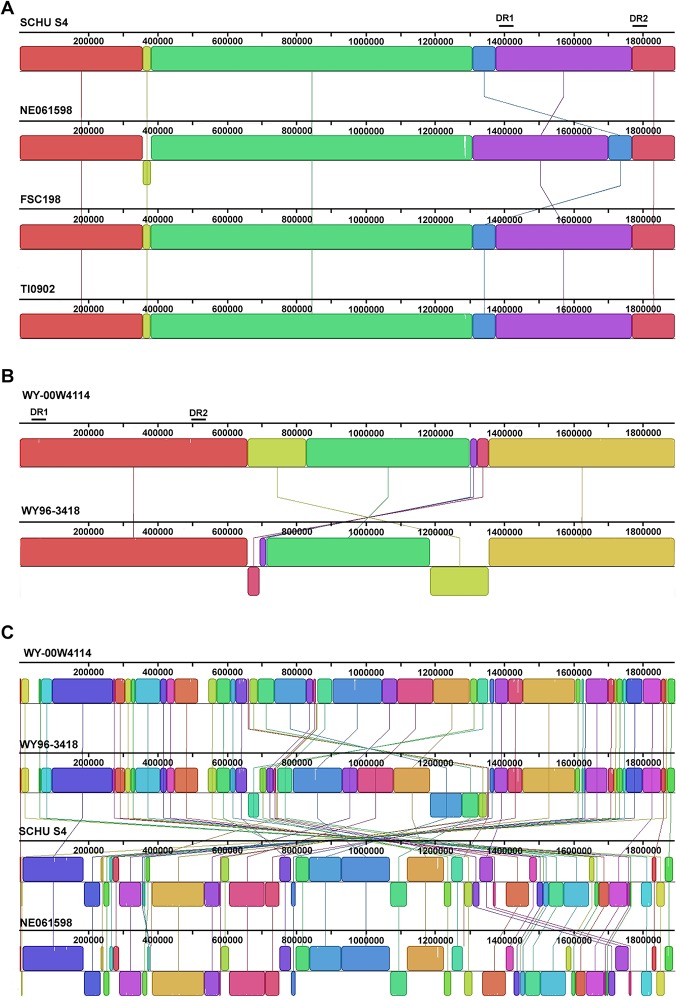
Genome alignment of *F*. *tularensis* A.I and A.II strains. Chromosomal alignments of representative *F*. *tularensis* A.I strains SCHU S4, NE061598, FSC198, and TI0902 (A); pairwise genome alignment of the *F*. *tularensis* A.II strains WY-00W4114 and WY96-3418 (B); and multiple chromosomal alignment of the *F*. *tularensis* A.I and A.II strains sequenced to completion and shown in panels A and B **(C)**. The relative location of the *Francisella* pathogenicity island (FPI) in duplicated region 1 (DR1) and duplicated region 2 (DR2) is identified with a bar above the associated chromosomal region for each subtype. The progressiveMauve software tool was used to align the genomes [21].

The spatial location of LCBs within the sequenced *F*. *tularensis* type A strains were compared; these analyses included the A.I strains SCHU S4, NE061598, FSC198, and TI0902 with WY96 and W4114 representing the A.II clade. Out of the four A.I genomes, only NE061598 differed in genomic organization due to one translocation and one inversion (Fig 4A). When comparing the chromosome of the two A.II isolates, four translocations and two inversions were apparent (Fig 4B). Additionally, a comparison between the *F*. *tularensis* A.I and A.II subpopulations revealed numerous differences in the spatial location of LCBs (Fig 4C), supporting the subdivision of these type A strains into at least two different clades.

### Comparison of interclade genomic content in *F*. *tularensis* A.I and A.II strains

The genomes of these *F*. *tularensis* A.I and A.II strains had 99.6% nucleotide identity to other members of their respective subpopulation, whereas a comparison between the A.I and A.II subtypes revealed 99.0–99.2% nucleotide identity. The circular chromosome of the *F*. *tularensis* A.I strains SCHU S4 and NE061598 and the A.II strains W4114 and WY96 were all approximately 1,890 kbp in size with a G+C content of 32.3% (Table 3). Moreover, all the completed A.I genomes (SCHU S4 GenBank accession number AJ749949, 1,892,775 bp; NE061598 GenBank accession number CP001633, 1,892,681 bp; FSC198 European Molecular Biology Laboratory accession number AM286280, 1,892,616 bp; and TI0902 GenBank accession number NC_016937, 1,892,744 bp) differed by less than 160 bp, and were isolated during a 63 year span from various geographical regions. The A.II strains were isolated within 4 years of each other from infected hosts in Wyoming and differed in chromosomal size by 776 bp (Table 3). Of note, the genomes of both A.II strains were approximately 6 kbp larger than the A.I clade, due in part to the presence of more IS elements (Tables 3 and 4).

**Table 3:** Overall genomic features of *F*. *tularensis* subtype A.I and A.II strains.

Feature	A.I		A.II	
	SCHU S4(1941)^a^	NE061598 (1998)^a^	WY96-3418 (1996)^a^	WY-00W4114 (2000)^a^
Length (bp)	1892775	1892681	1898476	1899252
GC Content (%)	32.3	32.3	32.3	32.3
Total ORFs	1852	1850	1872	1864
Disrupted ORFs/Pseudogenes	201	201	115	112
Large Duplicated Regions (>5 kbp)	3	3	3	3
*Francisella* Pathogenicity Island	2	2	2	2
Transposons (IS elements)^b^	66	66	71	71
Structural tRNA	38	38	38	38
Structural rRNA (3 operons)	10	10	10	10

^a^Year in parentheses denotes when the respective *F*. *tularensis* strain was isolated.

^b^Includes only full-length ORFs.

**Table 4:** Comparison of IS element content in *F*. *tularensis* A.I and A.II strains.

IS Elements	A.I^a^		A.II^a^	
	SCHU S4	NE061598	WY96-3418	WY-00W4114
IS*Ftu1* (IS630 family)	47 (3)	47 (3)	48 (8)	48 (8)
IS*Ftu2* (IS5 family)	13 (3)	13 (3)	18^b^ (18)	18^b^ (18)
IS*Ftu3* (ISNCY family, ISHpal-IS1016)	2 (1)	2 (1)	1 (3)	1 (3)
IS*Ftu4* (IS982 family)	1	1	1	1
IS*Ftu5* (IS4 family)	1	1	1	1
IS*Ftu6* (IS1595 family)	1 (1)	1 (1)	1 (1)	1 (1)
ISSod13 (IS3 family)	1	1	1	1
Total	66 (8)	66 (8)	71 (30)	71 (30)

^a^The total number of genes encoding full-length IS elements are shown and the number of IS element remnants are denoted in parentheses.

^b^IS *Ftu2* ORFs contain a premature stop codon.

The chromosome in both the *F*. *tularensis* A.I and A.II strains contained three large duplicated regions, in which DR1 and DR2 were considerably larger than DR3 due to the presence of a *Francisella* pathogenicity island (FPI). The 38 tRNA genes and three rRNA operons present in the sequenced A.I strains were also present in the genome of the A.II strains W4114 and WY96. However, a substantially higher abundance of disrupted genes or pseudogenes were present in the A.I subtype relative to the A.II subtype (Table 3).

The *F*. *tularensis* A.II strains contained more IS elements relative to the A.I strains, but also possessed a greater abundance of disrupted IS elements (Table 4). This disparity was predominantly due to the premature stop codon found in all IS*Ftu2* ORFs within the chromosome of the A.II strains. Gene decay of IS*Ftu1*, the most abundant transposase within both the A.I and A.II clade, was also more evident in the A.II strains (Table 4). The intact IS*Ftu1* genes in both the A.I and A.II chromosome contained two consecutive overlapping ORFs in which a programmed ribosomal frameshift is predicted to occur at the heptanucleotide AAAAAA, presumably resulting in the translation of a single functional protein with transposase activity [11,26]. One of the full-length IS*Ftu3* ORFs in the A.I subpopulation was apparently disrupted in the A.II strains, producing two IS*Ftu3* remnants in the A.II clade. No difference in copy number was observed between the A.I and A.II subtypes for IS*Ftu4*, IS*Ftu5*, IS*Ftu6*, and ISSod13.

Two of the three duplicated regions (DR1 and DR2) in the *F*. *tularensis* A.I and A.II strains were approximately 33.9 kbp in size. The third duplicated region (DR3) was considerable smaller in size (5.3 kbp) than DR1 and DR2, predominantly due to the absence of the FPI genes. In both the A.I and A.II subtypes, DR3 encoded five RNA structural genes, specifically 16S rRNA, tRNA-Ile, tRNA-Ala, 23S rRNA, and 5S rRNA that were located at nucleotides 849,941–855,477 in W4114. Although both the A.I and A.II strains have a duplicated FPI in DR1 and DR2, the spatial location of these regions differed between these two clades. In the A.II clade, DR1 and DR2 are located in reverse orientation on the chromosome relative to these regions in the A.I subpopulation (Fig 4A and 4B). In SCHU S4, DR1 and DR2 are located at nucleotide positions 1,374,371–1,408,281 and 1,767,715–1,801,625, respectively. In W4114, DR1 and DR2 are located at nucleotides 517,268–550,971 and 27,169–61,191, respectively, similar to the location of these regions in WY96. IS*Ftu1*, the most abundant IS element in both the A.I and A.II clades, flanks one end of DR1 and DR2 in both subpopulations, implicating a possible role in the initial acquisition of this pathogenicity island.

The *F*. *tularensis* A.II genomes contained 26 VNTR motifs of which 10 were absent in the A.I chromosome (S10 Table) [13,27]. The A.I genomes contained two VNTR sequences that were not in the A.II chromosome. The longest of these repetitive sequences in the A.II genomes were 60 nucleotides in length, whereas the longest VNTR in the A.I genomes was 23 nucleotides. In general, the chromosome of the A.II subtype contained more and longer VNTR motifs than the A.I genome.

When the sizes of DR1 and DR2 in the A.I strains SCHU S4 and NE061598 and A.II strains W4114 and WY96 were compared, DR2 in W4114 had multiple differences, whereas the lengths of the other duplicated regions were very similar. More specifically, DR2 in W4114 contained a 200 bp insertion in *pdpA* that would result in a truncated 62 residue protein, a 28 bp deletion just prior to the 16S rRNA gene, and a 60 bp deletion in the beginning of the 5S rRNA gene. W4114 DR2 also contained five nucleotide substitutions in the 16S rRNA ORF compared to the associated regions in the other A.II 16S rRNA genes. In DR1 and DR2 of the A.I strains, the 16S rRNA gene contained one nucleotide deletion relative to this gene in the A.II strains, and both the 16S and 23S rRNA genes contained a nucleotide specific to either the A.I or A.II clade. No other nucleotide polymorphisms were observed within the RNA structural ORFs in DR1 or DR2 of the *F*. *tularensis* A.I and A.II strains.

The 18 FPI genes within DR1 and DR2 were present in both the *F*. *tularensis* A.I and A.II strains, and included the intracellular growth locus *igl* genes and the pathogenicity determinant protein *pdp* genes. Many of the FPI products, particularly those encoded by the *igl and pdp* genes, have been determined to be critical for intramacrophage survival, replication, and virulence ([28] and review in [29]). The gene encoding PdpA in W4114 DR2 had a 200 bp insert that would result in a truncated 62 residue protein. This gene in DR1 of W4114, as well as the other A.II strain and the A.I strains encoded an 820 amino acid protein. A premature stop codon was previously reported for the WY96 *pdpC* genes in both DR1 and DR2 [12]. Our analyses did not identify a stop codon and therefore, showed no difference in the length of the predicted protein encoded by the *pdpC* genes in DR1 or DR2 for WY96, W4114, or the A.I strains. The *anmK* gene, which is predicted to encode an anhydro-N-acetylmuramic acid kinase within the FPI, contained a premature stop codon in DR1 and DR2 of the A.I strains and W4114 DR2, resulting in a truncated protein; however, in W4114 DR1 and WY96 DR1 and DR2 a 327 amino acid protein was predicted to be encoded. The remaining FPI genes in both loci of the A.I and A.II strains shared considerable nucleotide identity and were predicted to encode proteins that are identical in length.

Factors known to regulate the expression of the FPI genes were reported to be located elsewhere on the chromosome; these FPI regulatory proteins include the transcription factors MglA and SspA [30], as well as the orphan two-component members KdpD and PmrA [31,32]. The composition of MglA and PmrA were identical in the A.I and A.II strains, whereas comparison between the SspA and KdpD regulatory proteins revealed one and three residue differences, respectively. Together these findings indicated that although the *F*. *tularensis* A.I and A.II subtypes differ in virulence, the FPI gene products and the known regulatory factors were highly conserved.

## Discussion

### Considerable genomic plasticity exists in the *F*. *tularensis* A.II subtype compared to the A.I subtype

In this study, we sequenced and assembled the genome of *F*. *tularensis* subsp. *tularensis* subtype A.II strain (W4114) for comparison to the only other sequenced A.II strain (WY96) and to the fully sequenced genomes from subtype A.I. We have previously shown that IS elements provide a means to differentiate *F*. *tularensis* subtypes and subspecies [17]. The abundance of these ORFs along with other repetitive sequences, the numerous polymorphisms and rearrangements, and the inherent limitations of the current DNA sequencing methodologies made genome closure difficult. Nonetheless, accurate assembly of the W4114 contiguous sequences was achieved by a combination of whole genome mapping and application of computational tools, allowing for an in depth analysis that revealed important intraclade and interclade differences and similarities between the *F*. *tularensis* subtype A.I and A.II genomes.

A notable finding disclosed from this study was that the *F*. *tularensis* A.II subtype contains substantial genomic plasticity compared to the A.I subtype. The A.I strains collected over a 60 year period from different geographical regions shared considerable sequence conservation and chromosomal synteny [11–15]. In contrast, numerous polymorphisms were discovered between the *F*. *tularensis* A.II strains W4114 and WY96 isolated from infected hosts in Wyoming with only a four year separation in their acquisition. *F*. *tularensis* A.II strains have been isolated from both humans and animals, and the distribution of this clade is west of the 100th meridian in the United States [33]. Deletions and base pair substitutions appear to be a reliable means to determine phylogenetic relationships in *F*. *tularensis* [5,9], and the findings in this study may have application to forensic investigations in the future.

The genome of *F*. *tularensis* W4114 was determined to be 776 bp larger and have fewer disrupted ORFs than *F*. *tularensis* WY96, indicating that either WY96 has undergone more genome reduction and decay or alternatively, W4114 has acquired nucleotide content. The substantial number of polymorphisms observed between these A.II genomes suggests that they have been separately evolving. Numerous synonymous and nonsynonymous substitutions were also discovered between the W4114 and WY96 chromosomes that may serve as an indicator of selective pressure within a protein coding region. In addition, the intergenic mutations may also affect the regulation of the associated gene(s) and/or small RNA production. Whether these mutations were due to impaired DNA repair is unclear and will require additional study.

Mutational biases for C+G → A+T substitutions are common in bacteria [34]. These mutations result in A+T enrichment and often occur in intracellular bacteria due to reduced purifying selection [35]. A+T enrichment was observed in the *F*. *tularensis* A.II strain W4114 relative to the A.II strain WY96. Since the genome of *Francisella* has a high A+T content, codon usage and the availability of the appropriate tRNA may have contributed to the apparent A+T increase in the W4114 genome. Also of note, the majority of nucleotide substitutions in both of the *F*. *tularensis* A.II strains were transitions, specifically C to T and A to G. Although there are twice as many possible transversions, others have proposed that transitions occur more often in genomes due to the molecular mechanisms that generate them, such as oxidative deamination and tautomerization [34]. In particular, 5-methylcytosine has a high propensity to undergo spontaneous deamination to thymine and occurs about 10 times more often than other transitions [36].

### *F*. *tularensis* genomes in the A.I clade have more gene decay than in the A.II strains

Comparisons between the *F*. *tularensis* A.I and A.II genomes revealed a plethora of translocations. The A.I chromosome was consistently smaller and had considerably more disrupted ORFs than the genome of the A.II strains, indicating greater gene loss and decay within the A.I clade. There is compelling evidence that deletional bias is a major force that shapes bacterial genomes, particularly in host-associated bacteria due to decreased selection to maintain gene functionality [37]. The *F*. *tularensis* A.I subtype with considerably more disrupted genes than the A.II subtype may have retained the minimal genetic content required for niche survival, contributing to their apparent chromosomal stability.

### Chromosomal features and GC skew in *F*. *tularensis* A.I and A.II strains

In general, the size of the bacterial chromosome for a species remains moderately constant [37]. Consistent with this notion, the *F*. *tularensis* genome size has remained approximately the same, despite the genetic variation observed within the A.II clade and between the type A subpopulations. Macrodomain organization appears to be a major factor restricting chromosome plasticity [38]. Further, there is a general trend of C avoidance in the leading strand due to strand-biased spontaneous mutations, particularly C to T mutations from deamination [39]. Based on GC skew, the location of the *ter* region in the WY96 chromosome differed compared to W4114 and the A.I strains. This may be important since there is a strong tendency for the circular bacterial chromosome to maintain the *ori* and *ter* regions opposite of each other for physical balance during DNA replication [40]. The two replication forks need to reach the *ter* region at approximately the same time for optimal and synchronized completion of the bidirectional chromosomal replication. DNA rearrangements, as was found in the *F*. *tularensis* A.II strains (W4114 and WY96), may disrupt such a balance and promote additional chromosomal rearrangements.

### High number of repetitive sequences in *F*. *tularensis* A.II strains may promote additional genomic translocations

The presence of repeated sequences enhances the potential for homologous recombination that can lead to deletions, duplications, and/or genomic rearrangements and is a common feature of highly virulent pathogens such as *F*. *tularensis*, *Yersinia pestis*, *Burkholderia pseudomallei*, and *B*. *mallei*. Homologous recombination does not depend on the distance between repeat sequences, whereas illegitimate recombination is dependent on both the distance and length of the sequence [41]. In contrast to the *F*. *tularensis* A.I clade, the A.II subpopulation possesses a higher number of IS element remnants, providing “hot spots” for further IS-mediated recombination. The inverted repeats that typically define the transposon boundaries have the tendency to form secondary structures that can reduce DNA replication fidelity and increase genetic diversity [42,43]. Interestingly, although inverted repeats are more often deleted than amplified [44], most of the IS elements in both *F*. *tularensis* W4114 and WY96 have retained these flanking regions, which may promote more genomic translocations.

The presence of the numerous VNTR sequences in the *F*. *tularensis* chromosome also contributed to the genomic differences observed within this species. The addition or deletion of individual repeats to these repetitive elements is usually the result of a replication error or recombination event and leads to allelic variation. The presence of more and longer VNTR motifs in the genome of the A.II subtype compared to A.I subtype increases the probability for errors during DNA replication and could have contributed to the high number of polymorphisms observed between the A.II genomes. These repeated motifs along with the many intact and disrupted IS elements in the A.II strains could provide templates for homologous recombination, contributing to additional genomic alterations. Further, polymerase or ribosomal slippage can also occur at VNTR sequences, thereby reducing fidelity during the associated process and could provide a selective advantage.

The virulent *F*. *tularensis* subspecies *tularensis*, *holarctica*, and *mediasiatica* contain duplicated FPIs, unlike the opportunistic subspecies *novicida* and near neighbor *F*. *philomiragia* that contain only one FPI. The lower G+C content in the FPI relative to the remainder of the genome in *F*. *tularensis* indicates that this region was acquired by horizontal transfer [28]. Further, the presence of an IS*Ftu1* ORF flanking one end of both DR1 and DR2 raises intriguing questions as to the origin of these virulence genes. IS*Ftu1* belongs to the promiscuous IS*630-Tc1-mariner* superfamily of transposons that is found in numerous organisms, including arthropods, protozoans, animals, and humans [45,46]. Therefore, any of these possible hosts for *F*. *tularensis* or co-habiting organisms are a plausible source for the initial acquisition of this mobile element.

### Chromosomal location of duplicated *Francisella* pathogenicity islands differs between the A.I and A.II strains

Other studies have provided evidence that some of the FPI genes encode components of the type VI secretion system and that these gene products contribute to the ability of *F*. *tularensis* to subvert the host immune response ([47] and reviewed in [29,48]). Many bacterial species have been shown to utilize a type VI secretion system to translocate effectors into eukaryotic cells and modulate host immunity (reviewed in [49,50]). In the current study, a comparison of the duplicated FPI chromosomal location revealed that these loci were retained within members of the *F*. *tularensis* A.I and A.II subpopulations, but differed between these clades. However, the content of the FPI gene products and well-known regulatory factors in the A.I and A.II subtypes were highly conserved, with the exception of *pdpA* in DR2 of W4114 and *anmK*. These findings demonstrate that additional study is needed to elucidate the factors that contribute to the virulence differences noted between and within the *F*. *tularensis* A.I and A.II strains.

Pathogen evolution is influenced by abiotic and biotic environmental interactions, resulting in either an increase or decrease in virulence [51]. Higher genomic plasticity may prime conditions for the introduction or loss of virulence factors [52]. Genomic plasticity in the *F*. *tularensis* A.II clade and the resulting polymorphisms have apparently resulted in a pathogen with less virulence than the highly virulent A.I strains. We propose that pathoadaption, the retention of beneficial features in the context of infection contributes to the chromosomal stability noted for the A.I clade and favors a host-associated niche. The higher abundance of genes disrupted in the *F*. *tularensis* A.I subtype compared to the A.II subtype also supports the notion that A.I strains rely more heavily on host factors. In contrast, the A.II subpopulation appears to be evolving at a faster rate, perhaps due to the variable and extreme environment that this clade is exposed to and the impact this stress may have on the A.II strains and their hosts.

## Conclusion

The findings shown in this study demonstrate that *F*. *tularensis* subtype A.II contains a higher number of both intact genes and longer repetitive sequences than the A.I subtype. Considerable genetic variation was also noted within the A.II strains in contrast to the A.I clade. Further study is needed to determine the factors promoting *F*. *tularensis* genetic diversity and plasticity, and whether these features provide a fitness advantage.

## Supporting Information

S1 Fig Endonuclease restriction patterns reveal a novel *F*. *tularensis* subtype A.II strain. (PDF)

S1 Table Overall nucleotide composition differences within the *F*. *tularensis* A.II genomes of WY-00W4114 and WY96-3418. (PDF)

S2 Table Indel nucleotide identities within the *F*. *tularensis* A.II genomes of WY-00W4114 relative to WY96-3418. (PDF)

S3 Table Nucleotide substitution identities within the *F*. *tularensis* A.II genomes of WY-00W4114 relative to WY96-3418. (PDF)

S4 Table Number of indels within the *F*. *tularensis* A.II genomes of WY-00W4114 relative to WY96-3418. (PDF)

S5 Table Small indels (<10 base pairs) within the *F*. *tularensis* A.II genomes of WY-00W4114 relative to WY96-3418 and chromosomal location. (PDF)

S6 Table Large indels (≥10 base pairs) within the *F*. *tularensis* A.II genome of WY-00W4114 relative to WY96-3418 and chromosomal location. (PDF)

S7 Table Synonymous and nonsynonymous nucleotide substitutions within the *F*. *tularensis* A.II genomes of WY-00W4114 relative to WY96-3418 and chromosomal location. (PDF)

S8 Table ORFs disrupted by IS elements within the *F*. *tularensis* A.II chromosome of WY-00W4114. (PDF)

S9 Table ORFs disrupted by IS elements within the *F*. *tularensis* A.II chromosome of WY96-3418. (PDF)

S10 Table VNTR markers within the F. tularensis A.II genomes of WY-00W4114 and WY96-3416. (PDF)

## References

[1] Bhattacharjee Y. Biosecurity. Panel selects most dangerous select agents. Science. 2011;332: 1491–1492. 10.1126/science.332.6037.1491 21700845

[2] Centers for Disease Control and Prevention, Department of Health and Human Services. Possession, use, and transfer of select agents and toxins; final rule. Fed Regist. 2012;77: 61083–61115. 23038847

[3] Sjostedt A. Tularemia: history, epidemiology, pathogen physiology, and clinical manifestations. Ann N Y Acad Sci. 2007;1105: 1–29. 17395726 10.1196/annals.1409.009

[4] Oyston PC, Sjostedt A, Titball RW. Tularaemia: bioterrorism defence renews interest in *Francisella tularensis* . Nat Rev Microbiol. 2004;2: 967–978. 15550942 10.1038/nrmicro1045

[5] Larsson P, Elfsmark D, Svensson K, Wikstrom P, Forsman M, Brettin T, et al Molecular evolutionary consequences of niche restriction in *Francisella tularensis*, a facultative intracellular pathogen. PLoS Pathog. 2009;5: e1000472 10.1371/journal.ppat.1000472 19521508 PMC2688086

[6] Huber B, Escudero R, Busse HJ, Seibold E, Scholz HC, Anda P, et al Description of *Francisella hispaniensis* sp. nov., isolated from human blood, reclassification of *Francisella novicida* (Larson *et al*. 1955) Olsufiev *et al*. 1959 as *Francisella tularensis* subsp. *novicida* comb. nov. and emended description of the genus *Francisella* . Int J Syst Evol Microbiol. 2010;60: 1887–1896. 10.1099/ijs.0.015941-0 19783615

[7] Johansson A, Celli J, Conlan W, Elkins KL, Forsman M, Keim PS, et al Objections to the transfer of *Francisella novicida* to the subspecies rank of *Francisella tularensis* . Int J Syst Evol Microbiol. 2010;60: 1717–1718; author reply 1718–1720. 10.1099/ijs.0.022830-0 20688748 PMC7442299

[8] Molins CR, Delorey MJ, Yockey BM, Young JW, Sheldon SW, Reese SM, et al Virulence differences among *Francisella tularensis* subsp. *tularensis* clades in mice. PLoS One. 2010;5: e10205 10.1371/journal.pone.0010205 20419133 PMC2855709

[9] Birdsell DN, Johansson A, Ohrman C, Kaufman E, Molins C, Pearson T, et al *Francisella tularensis* subsp. *tularensis* group A.I, United States. Emerg Infect Dis. 2014;20: 861–865. 10.3201/eid2005.131559 24755401 PMC4012810

[10] Molins CR, Delorey MJ, Yockey BM, Young JW, Belisle JT, Schriefer ME, et al Virulence difference between the prototypic Schu S4 strain (A1a) and *Francisella tularensis* A1a, A1b, A2 and type B strains in a murine model of infection. BMC Infect Dis. 2014;14: 67 10.1186/1471-2334-14-67 24502661 PMC3923427

[11] Larsson P, Oyston PC, Chain P, Chu MC, Duffield M, Fuxelius HH, et al The complete genome sequence of *Francisella tularensis*, the causative agent of tularemia. Nat Genet. 2005;37: 153–159. 15640799 10.1038/ng1499

[12] Beckstrom-Sternberg SM, Auerbach RK, Godbole S, Pearson JV, Beckstrom-Sternberg JS, Deng Z, et al Complete genomic characterization of a pathogenic A.II strain of *Francisella tularensis* subspecies *tularensis* . PLoS One. 2007;2: e947 17895988 10.1371/journal.pone.0000947 PMC1978527

[13] Nalbantoglu U, Sayood K, Dempsey MP, Iwen PC, Francesconi SC, Barabote RD, et al Large direct repeats flank genomic rearrangements between a new clinical isolate of *Francisella tularensis* subsp. *tularensis* A1 and Schu S4. PLoS One. 2010;5: e9007 10.1371/journal.pone.0009007 20140244 PMC2815774

[14] Chaudhuri RR, Ren CP, Desmond L, Vincent GA, Silman NJ, Brehm JK, et al Genome sequencing shows that European isolates of *Francisella tularensis* subspecies *tularensis* are almost identical to US laboratory strain Schu S4. PLoS One. 2007;2: e352 17406676 10.1371/journal.pone.0000352 PMC1832225

[15] Modise T, Ryder C, Mane SP, Bandara AB, Jensen RV, Inzana TJ. Genomic comparison between a virulent type A1 strain of *Francisella tularensis* and its attenuated O-antigen mutant. J Bacteriol. 2012;194: 2775–2776. 10.1128/JB.00152-12 22535949 PMC3347185

[16] Dempsey MP, Nietfeldt J, Ravel J, Hinrichs S, Crawford R, Benson AK. Paired-end sequence mapping detects extensive genomic rearrangement and translocation during divergence of *Francisella tularensis* subsp. *tularensis* and *Francisella tularensis* subsp. *holarctica* populations. J Bacteriol. 2006;188: 5904–5914. 16885459 10.1128/JB.00437-06 PMC1540061

[17] Larson MA, Fey PD, Bartling AM, Iwen PC, Dempsey MP, Francesconi SC, et al *Francisella tularensis* molecular typing using differential insertion sequence amplification. J Clin Microbiol. 2011;49: 2786–2797. 10.1128/JCM.00033-11 21613430 PMC3147756

[18] Hesselbrock W, Foshay L. The morphology of *Bacterium tularense* . J Bacteriol. 1945;49: 209–231. 16560913 10.1128/jb.49.3.209-231.1945 PMC374032

[19] US Department of Health and Human Services. Biosafety in microbiological and biomedical laboratories 5th ed. Washington, DC: US Government Printing Office; 2007.

[20] Smith TF, Waterman MS. Identification of common molecular subsequences. J Mol Biol. 1981;147: 195–197. 7265238 10.1016/0022-2836(81)90087-5

[21] Darling AE, Mau B, Perna NT. progressiveMauve: multiple genome alignment with gene gain, loss and rearrangement. PLoS One. 2010;5: e11147 10.1371/journal.pone.0011147 20593022 PMC2892488

[22] Needleman SB, Wunsch CD. A general method applicable to the search for similarities in the amino acid sequence of two proteins. J Mol Biol. 1970;48: 443–453. 5420325 10.1016/0022-2836(70)90057-4

[23] Benson G. Tandem repeats finder: a program to analyze DNA sequences. Nucleic Acids Res. 1999;27: 573–580. 9862982 10.1093/nar/27.2.573 PMC148217

[24] Rohmer L, Fong C, Abmayr S, Wasnick M, Larson Freeman TJ, Radey M, et al Comparison of *Francisella tularensis* genomes reveals evolutionary events associated with the emergence of human pathogenic strains. Genome Biol. 2007;8: R102 17550600 10.1186/gb-2007-8-6-r102 PMC2394750

[25] Keim P, Johansson A, Wagner DM. Molecular epidemiology, evolution, and ecology of *Francisella* . Ann N Y Acad Sci. 2007;1105: 30–66. 17435120 10.1196/annals.1409.011

[26] Chandler M, Fayet O. Translational frameshifting in the control of transposition in bacteria. Mol Microbiol. 1993;7: 497–503. 8384687 10.1111/j.1365-2958.1993.tb01140.x

[27] Johansson A, Farlow J, Larsson P, Dukerich M, Chambers E, Bystrom M, et al Worldwide genetic relationships among *Francisella tularensis* isolates determined by multiple-locus variable-number tandem repeat analysis. J Bacteriol. 2004;186: 5808–5818. 15317786 10.1128/JB.186.17.5808-5818.2004 PMC516809

[28] Nano FE, Zhang N, Cowley SC, Klose KE, Cheung KK, Roberts MJ, et al A *Francisella tularensis* pathogenicity island required for intramacrophage growth. J Bacteriol. 2004;186: 6430–6436. 15375123 10.1128/JB.186.19.6430-6436.2004 PMC516616

[29] Broms JE, Sjostedt A, Lavander M. The Role of the *Francisella tularensis* pathogenicity island in type VI secretion, intracellular survival, and modulation of host cell signaling. Front Microbiol. 2010;1: 136 10.3389/fmicb.2010.00136 21687753 PMC3109350

[30] Brotcke A, Weiss DS, Kim CC, Chain P, Malfatti S, Garcia E, et al Identification of MglA-regulated genes reveals novel virulence factors in *Francisella tularensis* . Infect Immun. 2006;74: 6642–6655. 17000729 10.1128/IAI.01250-06 PMC1698089

[31] Mohapatra NP, Soni S, Bell BL, Warren R, Ernst RK, Muszynski A, et al Identification of an orphan response regulator required for the virulence of *Francisella* spp. and transcription of pathogenicity island genes. Infect Immun. 2007;75: 3305–3314. 17452468 10.1128/IAI.00351-07 PMC1932945

[32] Weiss DS, Brotcke A, Henry T, Margolis JJ, Chan K, Monack DM. *In vivo* negative selection screen identifies genes required for *Francisella* virulence. Proc Natl Acad Sci U S A. 2007;104: 6037–6042. 17389372 10.1073/pnas.0609675104 PMC1832217

[33] Kugeler KJ, Mead PS, Janusz AM, Staples JE, Kubota KA, Chalcraft LG, et al Molecular epidemiology of *Francisella tularensis* in the United States. Clin Infect Dis. 2009;48: 863–870. 10.1086/597261 19245342

[34] Ochman H. Neutral mutations and neutral substitutions in bacterial genomes. Mol Biol Evol. 2003;20: 2091–2096. 12949125 10.1093/molbev/msg229

[35] Balbi KJ, Rocha EP, Feil EJ. The temporal dynamics of slightly deleterious mutations in *Escherichia coli* and *Shigella* spp. Mol Biol Evol. 2009;26: 345–355. 10.1093/molbev/msn252 18984902

[36] Holliday R, Grigg GW. DNA methylation and mutation. Mutat Res. 1993;285: 61–67. 7678134 10.1016/0027-5107(93)90052-h

[37] Mira A, Ochman H, Moran NA. Deletional bias and the evolution of bacterial genomes. Trends Genet. 2001;17: 589–596. 11585665 10.1016/s0168-9525(01)02447-7

[38] Esnault E, Valens M, Espeli O, Boccard F. Chromosome structuring limits genome plasticity in *Escherichia coli* . PLoS Genet. 2007;3: e226 18085828 10.1371/journal.pgen.0030226 PMC2134941

[39] Marin A, Xia X. GC skew in protein-coding genes between the leading and lagging strands in bacterial genomes: new substitution models incorporating strand bias. J Theor Biol. 2008;253: 508–513. 10.1016/j.jtbi.2008.04.004 18486155

[40] Song J, Ware A, Liu SL. Wavelet to predict bacterial *ori* and *ter*: a tendency towards a physical balance. BMC Genomics. 2003;4: 17 12732098 10.1186/1471-2164-4-17 PMC156607

[41] Rocha EP. An appraisal of the potential for illegitimate recombination in bacterial genomes and its consequences: from duplications to genome reduction. Genome Res. 2003;13: 1123–1132. 12743022 10.1101/gr.966203 PMC403640

[42] Lin CT, Lin WH, Lyu YL, Whang-Peng J. Inverted repeats as genetic elements for promoting DNA inverted duplication: implications in gene amplification. Nucleic Acids Res. 2001;29: 3529–3538. 11522822 10.1093/nar/29.17.3529 PMC55881

[43] Voineagu I, Narayanan V, Lobachev KS, Mirkin SM. Replication stalling at unstable inverted repeats: interplay between DNA hairpins and fork stabilizing proteins. Proc Natl Acad Sci U S A. 2008;105: 9936–9941. 10.1073/pnas.0804510105 18632578 PMC2481305

[44] Zhao G, Chang KY, Varley K, Stormo GD. Evidence for active maintenance of inverted repeat structures identified by a comparative genomic approach. PLoS One. 2007;2: e262 17327921 10.1371/journal.pone.0000262 PMC1803023

[45] Shao H, Tu Z. Expanding the diversity of the *IS630-Tc1-mariner* superfamily: discovery of a unique DD37E transposon and reclassification of the DD37D and DD39D transposons. Genetics. 2001;159: 1103–1115. 11729156 10.1093/genetics/159.3.1103 PMC1461862

[46] Plasterk RH, Izsvak Z, Ivics Z. Resident aliens: the Tc1/*mariner* superfamily of transposable elements. Trends Genet. 1999;15: 326–332. 10431195 10.1016/s0168-9525(99)01777-1

[47] Broms JE, Meyer L, Lavander M, Larsson P, Sjostedt A. DotU and VgrG, core components of type VI secretion systems, are essential for *Francisella* LVS pathogenicity. PLoS One. 2012;7: e34639 10.1371/journal.pone.0034639 22514651 PMC3326028

[48] Dai S, Mohapatra NP, Schlesinger LS, Gunn JS. Regulation of *Francisella tularensis* virulence. Front Microbiol. 2010;1: 1–10. 10.3389/fmicb.2010.00001 21687801 PMC3109300

[49] Russell AB, Peterson SB, Mougous JD. Type VI secretion system effectors: poisons with a purpose. Nat Rev Microbiol. 2014;12: 137–148. 10.1038/nrmicro3185 24384601 PMC4256078

[50] Ho BT, Dong TG, Mekalanos JJ. A view to a kill: the bacterial type VI secretion system. Cell Host Microbe. 2014;15: 9–21. 10.1016/j.chom.2013.11.008 24332978 PMC3936019

[51] Pallen MJ, Wren BW. Bacterial pathogenomics. Nature. 2007;449: 835–842. 17943120 10.1038/nature06248

[52] Fraser-Liggett CM. Insights on biology and evolution from microbial genome sequencing. Genome Res. 2005;15: 1603–1610. 16339357 10.1101/gr.3724205

