# Supplementary material for: Francisella tularensis Subtype A.II Genomic Plasticity in Comparison with Subtype A.I
Source: PLoS One. 2015 Apr 28;10(4):e0124906. doi: 10.1371/journal.pone.0124906 (PMC4412822; doi:10.1371/journal.pone.0124906)

Additional file 1: Figure S1. Endonuclease restriction patterns reveal a novel *F. tularensis* subtype A.II strain. Comparison of PFGE with *Pme*I (left panel) and *Bam*HI (right panel) digestion patterns of *F. tularensis* WY-00W4114 (lanes 2 and 5) to two other A.II strains, specifically WY96-3418 (lanes 1 and 4) and WY-WPHL-06F12590 (lanes 3 and 6). *Pme*I and *Bam*HI restricted fragment polymorphisms are denoted with an arrow to the left and right, respectively. *Salmonella* serotype Braenderup strain (H9812) was used as the universal size standard, and the location of the resulting bands are denoted in kilobase pairs between the *Pme*I and *Bam*HI digestion patterns.

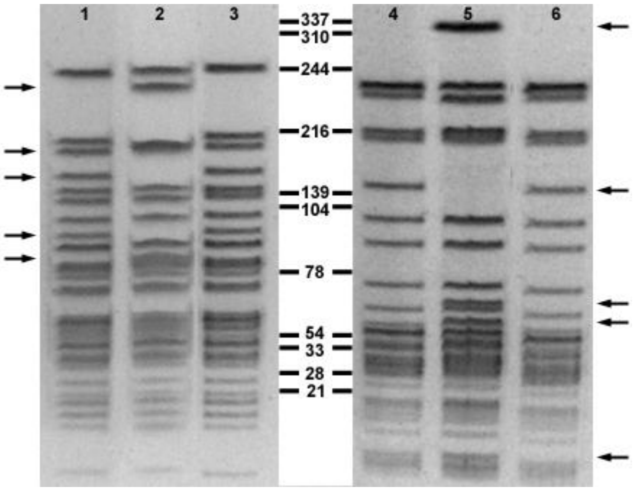

Supplement: S1 Fig — (PDF) [file pone.0124906.s001.pdf]
