# Supplementary material for: Francisella tularensis Subtype A.II Genomic Plasticity in Comparison with Subtype A.I
Source: PLoS One. 2015 Apr 28;10(4):e0124906. doi: 10.1371/journal.pone.0124906 (PMC4412822; doi:10.1371/journal.pone.0124906)
Supplement: S1 Table — (PDF) [file pone.0124906.s002.pdf]

Additional file 2: Table S1. Overall nucleotide composition differences within the *F. tularensis* A.II genomes of WY-00W4114 and WY96-3418.

	**WY-00W4114**	**WY96-3418**
**A**	641960	639661
**C**	302426	306937
**G**	310333	305675
**T**	644533	646203
**A+T Total**	1286493	1285864
**G+C Total**	612759	612612
**A+T Increase**	629	-
**G+C Increase**	147	-
