# Supplementary material for: Francisella tularensis Subtype A.II Genomic Plasticity in Comparison with Subtype A.I
Source: PLoS One. 2015 Apr 28;10(4):e0124906. doi: 10.1371/journal.pone.0124906 (PMC4412822; doi:10.1371/journal.pone.0124906)
Supplement: S2 Table — (PDF) [file pone.0124906.s003.pdf]

Additional file 3: Table S2. Indel nucleotide identities within the *F. tularensis* A.II genomes of WY-00W4114 relative to WY96-3418.

Indel	Inserted Nucleotide	Deleted Nucleotide
**A**	426	151
**C**	118	60
**G**	153	50
**T**	481	141
