# Supplementary material for: Francisella tularensis Subtype A.II Genomic Plasticity in Comparison with Subtype A.I
Source: PLoS One. 2015 Apr 28;10(4):e0124906. doi: 10.1371/journal.pone.0124906 (PMC4412822; doi:10.1371/journal.pone.0124906)
Supplement: S3 Table — (PDF) [file pone.0124906.s004.pdf]

Additional file 4: Table S3. Nucleotide substitution identities within the *F. tularensis* A.II genomes of WY-00W4114 relative to WY96-3418.

**Nucleotide Substitutions**			**WY96-3418** 145 A+T 141 G+C			
			**63**	**72**	**69**	**82**
			**A**	**C**	**G**	**T**
**WY-00W4114** 159 A+T 127 G+C	**73**	**A**	-	6	48	19
	**68**	**C**	13	-	5	50
	**59**	**G**	37	9	-	13
	**86**	**T**	13	57	16	-
