# Supplementary material for: Francisella tularensis Subtype A.II Genomic Plasticity in Comparison with Subtype A.I
Source: PLoS One. 2015 Apr 28;10(4):e0124906. doi: 10.1371/journal.pone.0124906 (PMC4412822; doi:10.1371/journal.pone.0124906)
Supplement: S4 Table — (PDF) [file pone.0124906.s005.pdf]

Additional file 5: Table S4. Number of indels within the *F. tularensis* A.II genomes of WY-00W4114 relative to WY96-3418.

Indel Size (bp)	Number of Occurrences
1	44
2	1
3	1
4	3
5	1
6	1
7	3
8	1
10	1
12	1
16	1
19	1
21	1
25	1
28	5
29	2
31	1
33	1
47	1
59	1
60	4
90	1
108	1
169	1
200	1
201	1
