# Supplementary material for: Francisella tularensis Subtype A.II Genomic Plasticity in Comparison with Subtype A.I
Source: PLoS One. 2015 Apr 28;10(4):e0124906. doi: 10.1371/journal.pone.0124906 (PMC4412822; doi:10.1371/journal.pone.0124906)
Supplement: S5 Table — (PDF) [file pone.0124906.s006.pdf]

Additional file 6: Table S5. Small indels (<10 base pairs) within the *F. tularensis* A.II genomes of WY-00W4114 relative to WY96-3418 and chromosomal location.

Polymorphism	WY-00W4114 5' Position	WY-00W4114 Sequence (5'→3')[a]	WY96-3418 Sequence (5'→3')[a]	Region (Product)
1 Nucleotide Position	26068	-	A	Intragenic (Metabolite:H+ symporter MHS family protein)
	227137	-	A	Intragenic (Glycerol kinase)
	252435	-	T	Intergenic
	550973	T	-	Intergenic
	703957	-	T	Intergenic
	830303	-	T	Intragenic (IS*Ftu*1)
	947571	-	C	Intergenic
	997269	A	-	Intergenic
	1094477	A	-	Intragenic (IS*Ftu*1)
	1205466	A	-	Intragenic (LysR transcriptional regulator)
	1305247	G	-	Intragenic (IS*Ftu*1)
	1358657	-	A	Intragenic (IS*Ftu*1)
	1359293	T	-	Intergenic
	1359332	T	-	Intergenic
	1359350	T	-	Intergenic
	1359363	G	-	Intergenic
	1359372	T	-	Intergenic
	1359385	C	-	Intergenic
	1359390	-	A	Intergenic[b]
	1359992	A	-	Intergenic[b]
	1359999	-	A	Intergenic
	1360041	C	-	Intergenic
	1360055	-	C	Intergenic
	1360071	-	T	Intergenic
	1360084	C	-	Intergenic
	1361369	A	-	Intragenic (IS*Ftu*1)
	1362012	A	-	Intergenic
	1391671	T	-	Intragenic (YjgP/YjgQ family permease)
	1607616	-	A	Intergenic
	1626637	A	-	Intergenic[b]
	1626644	-	A	Intergenic
	1626686	C	-	Intergenic
	1626700	-	C	Intergenic
	1626715	-	T	Intergenic

	1626729	C	-	Intergenic
	1628141	-	T	Intragenic (IS*Ftu*1)
	1636378	T	-	Intergenic
	1636391	-	A	Intergenic
	1636407	-	G	Intergenic
	1636420	G	-	Intergenic
	1636463	-	T	Intergenic
	1636470	T	-	Intergenic[b]
	1885946	-	T	Intergenic
	1897321	T	-	Intragenic (Ribonucleases G and E)
2 Nucleotide Positions	974624	--	CC	Intragenic (Hypothetical protein)
3 Nucleotide Positions	1852866	AAT	---	Intergenic
4 Nucleotide Positions	382558	TAGC	----	Intragenic (Hypothetical protein)
	1303248	CTGC	----	Intragenic (Peptidase M13 family)
	1636490	CTGG	----	Intergenic[b]
5 Nucleotide Positions	1675254	AAACA	-----	Intergenic
6 Nucleotide Positions	1093289	------	GGCTAA	Intragenic (L-lactate dehydrogenase)
7 Nucleotide Positions	866020	-------	TAATTGA	Intergenic
	1359934	CTTTTTG	-------	Intergenic[b]
	1626579	CTTTTTG	-------	Intergenic[b]
8 Nucleotide Positions	733938	TAAATATT	--------	Intergenic

[a]Indels were identified by aligning the corresponding and adjacent 5' and 3' flanking sequences in the genomes of WY-00W4114 and WY96-3418 and a dashed line indicates the absence of a nucleotide.

[b]NCBI annotation of WY-00W4114 genome predicts this region to contain a transposase remnant.
