# Supplementary material for: Francisella tularensis Subtype A.II Genomic Plasticity in Comparison with Subtype A.I
Source: PLoS One. 2015 Apr 28;10(4):e0124906. doi: 10.1371/journal.pone.0124906 (PMC4412822; doi:10.1371/journal.pone.0124906)
Supplement: S6 Table — (PDF) [file pone.0124906.s007.pdf]

Additional file 7: Table S6. Large indels (≥10 base pairs) within the *F. tularensis* A.II genomes of WY-00W4114 relative to WY96-3418 and chromosomal location.

Sequence (5' → 3')[a]	Type	Location in WY-00W4114	Region (Product)
CAGCTGGACA	Insertion	1066071-1066080	Intragenic (AhpC/TSA family peroxiredoxin)
ATTTTGATCATT	Deletion	1432348	Intragenic (Chitinase)
TGTAGAAAAAGATATT	Insertion	517290-517305	Intergenic
TAAACTTGAACACAAGTAC	Deletion	971106	Intragenic (Hypothetical protein)
AAGAGCAAGAAAGTTCACCAA	Insertion	482552-482572	Intragenic (ATP-dependent DNA helicase)
AGTGATAAATTTATAACAAATTATC	Deletion	969596	Intragenic (Hypothetical protein)
TAATCAGTAGTTAGAGTCAGAATTTGAG	Deletion	56028	Intergenic
TAATCAGTAGTTAGAGTCAGAATTTGAG	Deletion	850254	Intergenic
TTAGCCATTAAAGGGGAATTATCTAAAC	Insertion	1082233-1082260	Intergenic
TTTTAAGAATGATAAAATAATTGACCAG	Insertion	1359944-1359971	Intergenic[b]
TTTTAAGAATGATAAAATAATTGACCAG	Insertion	1626589-1626616	Intergenic[b]
TATAGTTAATCCGAAAGATATTTGTAGAA	Insertion	1325268-1325296	Intragenic (Hypothetical protein) and Intergenic
TTCTACAAATATCTTTCGGATTAACTATA	Deletion	229378	Intragenic (Glycerol-3-phosphate dehydrogenase)
ATTATTTTATCATTCTTAAAATCACAAAAAG	Insertion	1636497-1636527	Intergenic[b]
GATTCCAATCTGCGCAGTAATGACAGGTTTGGA	Insertion	424132-424164	Intergenic
AAATCTTTTTCTTAAAAAATTTTTTTAGCAATAATTTATTTAATTTT	Deletion	1725680	Intergenic
AATGTTATAATGTCTAATAAAAATGCCATCATATAGCCAATATTTTAGAGACATCGTAA	Deletion	334819	Intergenic
TATGCTGAATTGTTAAATATCTTTATAACCCAAAACACAGTTTTGGCGATGATAGCTTGT	Deletion	60766	Intragenic (5S ribosomal RNA)
AGTATTGTTATTGGTGAGCCTAGTTATGGTGAGGTTTTAGCATATCAAACACAAAGAAGA	Insertion	426324-426383	Intragenic (IS*Ftu*1)
TCAATGGTTTAGCAAATGAAGCTAAACCGCTGTTATTTAAGAGTTGAAAAGCAATAAATA	Insertion	1772331-1772390	Intergenic
ACTCGTTTAGCTCCAAAATAAGACTCATCTAACTCAAACTCCCCAGCAGACGCAAAGAAT	Deletion	1885897	Intergenic
TATTATCCTTATTATCCTTATTATCCTTATTATCCTTATTATCCTTATTATCCTTATTATCCTTATTATCCTTATTATCCTTATTGTCTT	Insertion	1683662-1683751	Intragenic (Hypothetical protein)

TATATTTATATTTATATTTATATTTAT<br>ATTTATATTTATATTTATATTTATATT<br>TATATTTATATTTATATTTATATTTAT<br>ATTTATATTTATATTTATATTTATATT	Insertion	1893213-1893320	Intragenic<br>(Hypothetical protein)
AAGTATTAATTATAAGTATTAATTAT<br>AAGTATTAATTATAAGTATTAATTAT<br>AAGTATTAATTATAAGTATTAATTAT<br>AAGTATTAATTATAAGTATTAATTAT<br>AAGTATTAATTATAAGTATTAATTAT<br>AAGTATTAATTATAAGTATTAATTAT<br>AAGTATTAATTAT	Insertion	830604-830772	Intergenic
CTGTTGCAGGAGTTATACAAGTTAA<br>GTTATCTAAGTATTCAGATAAAAAAA<br>CTTTAGTAGCTCGAGTATCAGAGAG<br>AGAAGAGTTTTTACAAGAAGTAGTT<br>GATAATGCTAATGAATTTATAGGCT<br>TTGCTTATAATGATTTATTTTTGCCC<br>AATACAGAAGGCAAGCTTTATTGTT<br>CAGAACTAATTCATGCTGCTTTTT	Insertion	494432-494632	Intragenic<br>(Hypothetical protein)

[a]Indels were identified by aligning the corresponding and adjacent 5' and 3' flanking sequences in the genomes of WY-00W4114 and WY96-3418.

[b]NCBI annotation of WY-00W4114 genome predicts this region to contain a transposase remnant.
