# Supplementary material for: Francisella tularensis Subtype A.II Genomic Plasticity in Comparison with Subtype A.I
Source: PLoS One. 2015 Apr 28;10(4):e0124906. doi: 10.1371/journal.pone.0124906 (PMC4412822; doi:10.1371/journal.pone.0124906)
Supplement: S7 Table — (PDF) [file pone.0124906.s008.pdf]

Additional file 8: Table S7. Nucleotide substitutions within the *F. tularensis* A.II genomes of WY-00W4114 relative to WY96-3418 and chromosomal location.

Position in WY-00W4114	WY-00W4114 Nucleotide	WY96-3418 Nucleotide	Residue Change[a,b]	Gene Product or Intergenic Region
3822	T	C	D → G	Transposase (IS*Ftu*1)[e]
14407	A	T	Synonymous	AAA ATPase
27401	A	G	D → G	Transposase (IS*Ftu*1)[c]
29521	A	T	Y → Stop	Anhydro-N-acetylmuramic acid kinase[c]
41709	C	T	V → I*	Pathogenicity determinant protein PdpC2[c]
55867	T	A		Intergenic[c]
55870	C	A		Intergenic[c]
56040	T	G		16S ribosomal RNA[c]
56058	T	G		16S ribosomal RNA[c]
56113	T	G		16S ribosomal RNA[c]
56123	T	G		16S ribosomal RNA[c]
56124	G	A		16S ribosomal RNA[c]
62132	A	G	D → G	Transposase (IS*Ftu*1)
62236	G	A	D → N	Transposase (IS*Ftu*1)
73023	T	C	V → A	Tryptophan/tyrosine permease family protein
86390	T	G	V → G	Glutamate decarboxylase
93328	A	G	N → D	Transposase (IS*Ftu*1)
110029	A	G	Synonymous	Multidrug resistance transporter, Bcr/CflA family
110032	T	G	Synonymous	Multidrug resistance transporter, Bcr/CflA family
128059	C	G	L → V*	NADH dehydrogenase I, M subunit
128238	C	T	Synonymous	NADH dehydrogenase I, M subunit
142857	T	C	Synonymous	Ribosomal large subunit pseudouridine synthase C
176071	G	A	G → S	Aconitate hydratase
187475	C	T	D → N	Transposase (IS*Ftu*1)[e]
187703	A	T		Intergenic
187704	T	A		Intergenic
187706	A	T		Intergenic
187727	C	A		Intergenic
187781	T	G		Intergenic
228161	A	G	Synonymous	Transposase (IS*Ftu*1)
233307	T	A	D → E*	Putative ATP-dependent exoDNase
245355	A	G	Synonymous	DNA-directed RNA polymerase beta subunit
270786	G	A	D → N	Transposase (IS*Ftu*1)
271267	C	T	Synonymous	Transposase (IS*Ftu*1)
275741	A	G	Synonymous	Transposase (IS*Ftu*1)
276326	C	T	G → D	Transposase (IS*Ftu*1)
278941	T	C	I → T	Hypothetical protein
306028	T	C	N → D	Transposase (IS*Ftu*1)
309581	T	C	I → V*	Bifunctional 3-demethylubiquinone-9 3-methyltransferase/ 2-octaprenyl-6-hydroxy phenol methylase
324585	C	T	E → K	Hypothetical protein
335052	A	G	N → D	Transposase (IS*Ftu*1)[e]
336490	T	G	S → A	Adenine specific DNA methylase

340614	A	C	D → A	Restriction endonuclease
358437	G	A	H → Y	UDP-galactose-lipid carrier transferase
364942	A	G		Intergenic
366565	A	G	Y → C	Ribonuclease III
413983	T	C	L → P	NAD dependent epimerase
467530	A	G	N → D	Membrane protein
481912	G	A	G → S	ATP-dependent DNA helicase
491631	A	G	S → P	Hypothetical protein
495247	T	C	N → D	Transposase (IS*Ftu*1)
503517	T	G	Y → S	Acyl carrier protein
517500	A	G	D → G	Transposase (IS*Ftu*1)[c]
557672	C	T	Synonymous	PEP-dependent sugar phosphotransferase system (PTS) family protein
571396	C	T	Synonymous	Transposase (IS*Ftu*1)
599528	T	A	D → E*	Guanosine-3', 5'-bis(diphosphate) 3'-pyrophosphohydrolase/(p)ppGpp synthase
599552	C	T	Synonymous	Guanosine-3', 5'-bis(diphosphate) 3'-pyrophosphohydrolase/(p)ppGpp synthase
599564	A	C	K → N	Guanosine-3', 5'-bis(diphosphate) 3'-pyrophosphohydrolase/(p)ppGpp synthase
600635	T	G	N → K	Guanosine-3', 5'-bis(diphosphate) 3'-pyrophosphohydrolase/(p)ppGpp synthase
610791	G	A	G → D	Transposase (IS*Ftu*1)
625850	T	G		Intergenic
629448	C	A		Intergenic
635958	T	C	S → G	Organic solvent tolerance protein precursor
649226	T	C	Y → H	Cation-efflux family protein
658404	A	G	D → G	Transposase (IS*Ftu*1)
658508	G	A	D → N	Transposase (IS*Ftu*1)
695087	C	T	A → V	Betanie/carnitine/choline transporter BCCT family protein
695480	A	T	Synonymous	Tryptophan-rich sensory protein
732724	T	C	L → P	Hypothetical protein
738130	G	A	D → N	Transposase (IS*Ftu*1)
738611	C	T	Synonymous	Transposase (IS*Ftu*1)
757836	C	T	Synonymous	Deoxyribodipyrimidine photolyase
764284	G	A		tRNA-Ser
775237	T	G		Intergenic
776127	G	T	S → Y	Heavy metal cation transport ATPase
780137	T	C	I → V*	Hypothetical protein
824797	T	C	S → P	Malate dehydrogenase
838487	A	G		Intergenic
843254	A	T	F → I*	Hypothetical protein
861233	T	G		Intergenic
864783	T	C	F → L	Amino acid transporter
896600	T	C	T → A	Tryptophanyl-tRNA synthetase
899442	A	G	K → E	Pyruvate dehydrogenase subunit E1
929384	G	T	P → Q	2-amino-4-hydroxy-6-hydroxymethyldihydropteridine pyrophosphokinase/dihydropteroate synthase
943771	A	G	Synonymous	Glutathione reductase
947524	G	T		Intergenic
969105	T	C	V → A	Hypothetical protein
993663	T	C	I → M*	Hypothetical protein
1014668	G	T	Synonymous	Inorganic pyrophosphatase
1016763	A	G	Y → C	Putative lipoate regulatory protein YbeD

1031631	C	T	Synonymous	FKBP-type peptidyl-prolyl cis-trans isomerase FklB
1041437	T	C	N → D	Ribosomal-protein-alanine acetyltransferase
1052051	T	C	V → A	3-phosphoserine/phosphohydroxythreonine aminotransferase
1057866	A	T	N → K	NAD-dependent aldehyde dehydrogenase
1080695	T	C	Synonymous	Hypothetical protein
1093935	G	A	D → N	Transposase (IS*Ftu*1)[e]
1094416	C	T	Synonymous	Transposase (IS*Ftu*1)[e]
1101180	A	C	I → L*	Amino acid transporter
1120183	A	G	S → P	Isochorismatase hydrolase family protein
1136459	C	T	Synonymous	Hypothetical protein
1150139	T	C	Q → R	Low molecular weight protein tyrosine phosphatase
1156530	T	C	I → M*	Hypothetical protein
1158481	G	A	A → V	DNA-binding, ATP-dependent protease La
1197473	G	T		Intergenic
1206122	T	C	D → G	Queuine tRNA-ribosyltransferase
1211525	G	T	M → I*	ABC transporter permease
1225648	T	C	Synonymous	Hypothetical protein
1228453	G	A	Synonymous	Deoxyribodipyrimidine photolyase
1249590	C	T	Synonymous	Adenylate kinase
1266917	A	G		Intergenic
1274294	T	A	E → D*	Gamma-glutamyltransferase
1305253	C	T	D → N	Transposase (IS*Ftu*1)[e]
1305357	C	T	G → D	Transposase (IS*Ftu*1)[e]
1326085	C	T	Synonymous	Transposase (IS*Ftu*1)[e]
1326925	A	G	I → T	Di or tripeptide permease YjdL
1351001	G	A	G → D	Long chain fatty acid CoA ligase
1359386	T	C		Intergenic
1359868	T	C		Intergenic[d]
1359875	C	A		Intergenic[d]
1359877	C	A		Intergenic[d]
1359881	A	G		Intergenic[d]
1359889	C	T		Intergenic[d]
1359892	C	T		Intergenic[d]
1359893	A	T		Intergenic[d]
1359898	A	G		Intergenic[d]
1359899	C	T		Intergenic[d]
1359905	T	C		Intergenic[d]
1359908	T	C		Intergenic[d]
1359911	G	A		Intergenic[d]
1359912	G	T		Intergenic[d]
1359913	C	T		Intergenic[d]
1359924	G	C		Intergenic[d]
1359928	G	C		Intergenic[d]
1360000	G	C		Intergenic
1360006	C	T		Intergenic
1360007	C	A		Intergenic
1360008	A	T		Intergenic
1360011	G	A		Intergenic
1360012	A	T		Intergenic
1360018	A	G		Intergenic
1360019	T	A		Intergenic
1360032	C	T		Intergenic
1360038	T	C		Intergenic
1360044	C	T		Intergenic

1360050	A	C		Intergenic
1360063	A	T		Intergenic
1360064	T	C		Intergenic
1360072	G	C		Intergenic
1360076	T	C		Intergenic
1360079	G	A		Intergenic
1360083	A	T		Intergenic
1362061	T	C	D → G	Transposase (IS*Ftu*1)[e]
1362134	G	C	H → D	Transposase (IS*Ftu*1)[e]
1376172	C	T	D → N	Transposase (IS*Ftu*1)
1399444	C	A	G → V	Fe-S protein-like protein of lactate dehydrogenase
1414955	C	A		Intergenic
1443940	G	A	Synonymous	Putative ROK-family transcriptional regulator
1458580	C	T	Synonymous	Transposase (IS*Ftu*1)
1480142	A	G	D → G	Transcriptional regulator
1480247	C	T	T → I	Transcriptional regulator
1490634	G	A	V → I*	GTPase
1523124	A	G	Synonymous	Cell division protein FtsW
1526842	C	A	Synonymous	Phosphoenolypyruvate carboxykinase [ATP]
1536330	T	C	T → A	Hypothetical protein
1543609	C	T	Synonymous	N-carbamoylputrescine amidase
1553400	A	G		Intergenic
1554494	C	T	Synonymous	Aspartate-semialdehyde dehydrogenase
1595614	G	T	Synonymous	Topoisomerase IV subunit A
1607606	T	A		Intergenic
1609705	A	G	D → G	Transposase (IS*Ftu*1)
1619458	A	G	Synonymous	Multifunctional nucleoside diphosphate kinase/apyrimidinic endonuclease/3'-phosphodiesterase
1625727	C	T	Synonymous	Transposase (IS*Ftu*1)
1625837	A	G	N → S	Transposase (IS*Ftu*1)
1626034	T	A		Intergenic[d]
1626513	T	C		Intergenic[d]
1626520	C	A		Intergenic[d]
1626522	C	A		Intergenic[d]
1626526	A	G		Intergenic[d]
1626534	C	T		Intergenic[d]
1626537	C	T		Intergenic[d]
1626538	A	T		Intergenic[d]
1626543	A	G		Intergenic[d]
1626544	C	T		Intergenic[d]
1626550	T	C		Intergenic[d]
1626553	T	C		Intergenic[d]
1626556	G	A		Intergenic[d]
1626557	G	T		Intergenic[d]
1626558	C	T		Intergenic[d]
1626569	G	C		Intergenic[d]
1626573	G	C		Intergenic[d]
1626645	G	C		Intergenic
1626651	C	T		Intergenic
1626652	C	A		Intergenic
1626653	A	T		Intergenic
1626656	G	A		Intergenic
1626657	A	T		Intergenic
1626663	A	G		Intergenic
1626664	T	A		Intergenic

1626677	C	T		Intergenic
1626683	T	C		Intergenic
1626689	C	T		Intergenic
1626695	A	C		Intergenic
1626708	A	T		Intergenic
1626709	T	C		Intergenic
1626717	G	C		Intergenic
1626721	T	C		Intergenic
1626724	G	A		Intergenic
1626728	A	T		Intergenic
1628119	A	G	Synonymous	Transposase (IS*Ftu*1)[e]
1628599	C	T	D → N	Transposase (IS*Ftu*1)[e]
1634506	G	A	D → N	Transposase (IS*Ftu*1)
1634987	T	C	Synonymous	Transposase (IS*Ftu*1)
1636377	G	A		Intergenic
1636382	C	T		Intergenic
1636385	A	G		Intergenic
1636389	C	G		Intergenic
1636397	A	G		Intergenic
1636398	T	A		Intergenic
1636411	T	G		Intergenic
1636417	G	A		Intergenic
1636423	A	G		Intergenic
1636429	G	A		Intergenic
1636442	A	T		Intergenic
1636443	T	C		Intergenic
1636449	T	A		Intergenic
1636450	C	T		Intergenic
1636453	T	A		Intergenic
1636454	G	T		Intergenic
1636455	G	A		Intergenic
1636461	C	G		Intergenic
1636533	C	G		Intergenic[d]
1636537	C	G		Intergenic[d]
1636548	G	A		Intergenic[d]
1636549	C	A		Intergenic[d]
1636550	C	T		Intergenic[d]
1636553	A	G		Intergenic[d]
1636556	A	G		Intergenic[d]
1636562	G	A		Intergenic[d]
1636563	T	C		Intergenic[d]
1636568	T	A		Intergenic[d]
1636569	G	A		Intergenic[d]
1636572	G	A		Intergenic[d]
1636580	T	C		Intergenic[d]
1636584	G	T		Intergenic[d]
1636586	G	T		Intergenic[d]
1636593	A	G		Intergenic[d]
1637072	A	T		Intergenic[d]
1637269	T	C	Synonymous	Transposase (IS*Ftu*1)
1637379	G	A	N → S	Transposase (IS*Ftu*1)
1640537	G	A	Synonymous	Transposase (IS*Ftu*1)
1641018	T	C	N → D	Transposase (IS*Ftu*1)
1643057	T	C	I → V*	30S ribosomal protein S4
1676205	G	T	Synonymous	Hypothetical protein
1681291	C	T	V → I*	Pyrrolidone-carboxylate peptidase
1682444	T	C	Synonymous	Hypothetical protein

1697947	A	G	R → G	Major facilitator superfamily (MFS) transport protein
1699849	G	A	G → D	Cytochrome d terminal oxidase, polypeptide subunit I
1704070	A	G	Synonymous	Transposase (IS*Ftu*1)
1704551	C	T	D → N	Transposase (IS*Ftu*1)
1707093	T	C	Synonymous	LpsA protein
1715739	G	A	Synonymous	ABC-type nitrate/sulfonate/bicarbonate transport system, ATPase component
1716984	T	G	C → G	Hypothetical protein
1722616	A	G	D → G	Transposase (IS*Ftu*1)[e]
1722720	G	A	D → N	Transposase (IS*Ftu*1)[e]
1730528	T	C	Y → C	Transposase (IS*Ftu*1)
1731095	T	C	N → D	Transposase (IS*Ftu*1)
1736252	C	T	E → K	Hypothetical protein
1747578	G	A	G → D	Transposase (IS*Ftu*1)[e]
1757511	C	T	D → N	Transposase (IS*Ftu*1)
1765723	T	C	Synonymous	Zinc ABC transporter, ATP-binding protein ZnuC
1766622	A	G	T → A	Zinc ABC transporter inner membrane permease protein ZnuB
1767536	T	G		Intergenic
1769230	A	C	Synonymous	Adenylosuccinate synthetase
1769232	C	T	D → K	Adenylosuccinate synthetase
1772298	C	T		Intergenic
1772301	T	C		Intergenic
1805363	A	G	Synonymous	Transposase (IS*Ftu*1)
1814522	A	G	N → D	Major facilitator transporter
1817153	T	C	Synonymous	Phosphoheptose isomerase 1
1827535	A	G	Synonymous	Transposase (IS*Ftu*1)
1828120	T	C	D → G	Transposase (IS*Ftu*1)
1858656	T	C	D → G	Transposase (IS*Ftu*1)[e]
1858760	T	C	N → D	Transposase (IS*Ftu*1)[e]
1871661	C	T	G → D	Transposase (IS*Ftu*1)[e]
1876392	A	G	N → D	Transposase (IS*Ftu*1)
1895214	T	C	I → V*	Anthranilate synthase component I

[a]Nucleotide substitutions located in coding regions are either indicated as synonymous or nonsynonymous, and if nonsynonymous, the resulting amino acid change is designated using the standard 1-letter code.

[b]An asterisk denotes a conservative nonsynonymous residue change with similar physiochemical properties.

[c]Nucleotide substitution is located within a duplicated region in the chromosome.

[d]NCBI annotation predicts this region to contain a transposase remnant.

[e]NCBI annotation predicts this region to contain a transposase even though numerous indels and substitutions are present.
