# Supplementary material for: Francisella tularensis Subtype A.II Genomic Plasticity in Comparison with Subtype A.I
Source: PLoS One. 2015 Apr 28;10(4):e0124906. doi: 10.1371/journal.pone.0124906 (PMC4412822; doi:10.1371/journal.pone.0124906)
Supplement: S8 Table — (PDF) [file pone.0124906.s009.pdf]

Additional file 9: Table S8. ORFs disrupted by IS elements within the *F. tularensis* A.II chromosome of WY-00W4114.

Locus tag	Position	Product
FT4114_00015	Complement (2692-3096)	Metabolite:H+ symporter family protein
FT4114_00030	Complement (4092-4616)	Nicotinamide ribonucleoside uptake permease family protein PnuC
FT4114_00175	Complement (28116-28994)	Fucose permease
FT4114_00280	61192-61908	NAD-dependent formate dehydrogenase
FT4114_00295	62871-63290	Formate dehydrogenase
FT4114_00420	94088-94450	DNA-cytosine methyltransferase
FT4114_00895	186342-186887	Hypothetical protein
FT4114_00940	Complement (191717-192298)	Hypothetical protein
Not assigned	Complement (226528-228035)	Glycerol kinase
Not assigned	270023-270525	Methyltransferase RlmH
FT4114_01525	Complement (305110-305340)	Hypothetical protein
FT4114_01610	322558-323481	Hypothetical protein
FT4114_01900	Complement (373050-373736)	Cyclopropane-fatty-acyl-phospholipid synthase
FT4114_01915	Complement (374612-375163)	Cyclopropane-fatty-acyl-phospholipid synthase
FT4114_02385	Complement(462586-462756)	Hypothetical protein
FT4114_02530	494404-494640	Hypothetical protein
FT4114_02545	495508-495777	Hypothetical protein
FT4114_02675	Complement (516929-517102)	Fucose permease-like protein
FT4114_02690	Complement (518215-519093)	Fucose permease
FT4114_03130	611477-611749	Hypothetical protein
FT4114_04130	Complement (774492-775190)	Heavy metal cation transport ATPase
FT4114_04145	Complement (776018-777520)	Heavy metal cation transport ATPase
FT4114_04515	844168-844782	Sua5/YciO/YrdC family protein
FT4114_05650	1048905-1049960	Histidine acid phosphatase
Not assigned	1050812-1051063	Intergenic (histidine acid phosphatase[a])
FT4114_05665	1051138-1052190	3-Phosphoserine/phosphohydroxythreonine aminotransferase
FT4114_06680	1229701-1229826	Hypothetical protein
FT4114_06695	1230777-1230956	Hypothetical protein
FT4114_08265	Complement (1538716-1538904)	rRNA methylases
FT4114_08280	Complement (1539756-1540412)	rRNA methylases
FT4114_09010	Complement(1668558-1669535)	Hypothetical protein
FT4114_09190	1703826-1703963	Major facilitator transporter
FT4114_09310	1722990-1723259	Hypothetical protein
FT4114_09350	Complement (1729451-1730419)	Major facilitator permease
FT4114_09365	Complement (1731427-1731609)	Metabolite:H+ symporter family protein
FT4114_09460	1747194-1747388	Hypothetical protein
FT4114_09475	1747952-1748425	Hypothetical protein
FT4114_09510	1757772-1757927	Ribosomal RNA large subunit methyltransferase H
FT4114_09610	Complement (1773141-1773350)	Serine permease
FT4114_09885	1826814-1827428	Na+/H+ antiporter
Not assigned	Complement (1828550-1828720)	Intergenic (hypothetical protein[a])
FT4114_09900	Complement (1829271-1830494)	Amino acid transporter
FT4114_10030	Complement(1856674-1858386)	Glycosyl hydrolase family protein
FT4114_10130	Complement (1871851-1872186)	Group 1 glycosyl transferase
FT4114_10220	Complement (1885064-1885207)	Aminopeptidase N
Not assigned	Complement (1886147-1888713)	Membrane alanine aminopeptidase N

[a]Annotation of corresponding allele in WY96-3418 is denoted in parentheses.
