# Supplementary material for: Francisella tularensis Subtype A.II Genomic Plasticity in Comparison with Subtype A.I
Source: PLoS One. 2015 Apr 28;10(4):e0124906. doi: 10.1371/journal.pone.0124906 (PMC4412822; doi:10.1371/journal.pone.0124906)
Supplement: S9 Table — (PDF) [file pone.0124906.s010.pdf]

Additional file 10: Table S9. ORFs disrupted by IS elements within the *F. tularensis* A.II chromosome of WY96-3418.

Locus tag	Position	Product
FTW_0003	Complement (2692-3096)	MFS transporter protein
FTW_0006	Complement (4092-4616)	Putative nicotinamide mononucleotide uptake protein
FTW_0035	Complement (28117-28995)	Putative membrane transporter
FTW_0055	61033-61797	NAD dependent formate dehydrogenase
FTW_0058	62760-63179	NAD dependent formate dehydrogenase
FTW_0083	93977-94339	Restriction endonuclease FnuDI
FTW_0179	186231-186776	Hypothetical protein
FTW_0188	Complement (191606-192187)	Hypothetical protein
FTW_0219	226417-227925	Glycerol kinase GlpD
FTW_0261	Complement (269914-270225)	Conserved hypothetical protein
FTW_0302	Complement (305001-305231)	Hypothetical protein
FTW_0320	322452-323372	Hypothetical protein
FTW_0379	Complement (373000-373686)	Cyclopropane-fatty-acyl-phospholipid synthase
FTW_0382	Complement (374562-375113)	Cyclopropane-fatty-acyl-phospholipid synthase
FTW_0473	Complement (462439-462609)	Hypothetical protein
Not assigned	494057-494271	Intergenic (hypothetical protein[a])
FTW_0504	495142-495408	Hypothetical protein
FTW_0529	Complement (516560-516955)	Major facilitator family transporter
FTW_0532	Complement (517830-518708)	Major facilitator family transporter
Not assigned	611091-611363	Intergenic (hypothetical protein[a])
FTW_0755	729071-729685	Sua5 family protein
FTW_0976	933889-934944	Histidine acid phosphatase
FTW_0979	935796-936047	Histidine acid phosphatase
FTW_0980	Complement (936122-937174)	SerC
FTW_1176	1114651-1114776	Hypothetical protein
FTW_1179	1115628-1115906	Hypothetical protein
FTW_1315	1242392-1243894	P-type ATPase
FTW_1318	1244722-1245420	P-type ATPase
FTW_1631	Complement (1538167-1538358)	RNA methyltransferase
FTW_1634	Complement (1539207-1539863)	RNA methyltransferase, TrmH family
FTW_1777	Complement (1667941-1668918)	Hypothetical protein
FTW_1834	1722362-1722547	Hypothetical protein
FTW_1842	Complement (1728786-1729754)	Major facilitator family transporter
FTW_1845	Complement (1730762-1730944)	Major facilitator superfamily transporter
FTW_1862	1746529-1746723	Hypothetical protein
FTW_1865	1747389-1747760	Hypothetical protein
Not assigned	1757107-1757262	Intergenic (ribosomal RNA large subunit methyltransferase H[a])
FTW_1891	Complement (1772416-1772625)	Membrane transporter family protein
FTW_1947	1826089-1826703	Na+/H+ antiporter
FTW_1950	1827825-1827995	Hypothetical protein
FTW_1951	Complement (1828546-1829769)	Amino acid transport
FTW_1977	Complement (1855946-1857658)	Glycosyl hydrolase family 32 protein
FTW_1996	Complement (1871123-1871458)	Glycosyl transferase
FTW_2012	Complement (1884336-1884479)	Hypothetical protein
FTW_2014	Complement (1885480-1888046)	Aminopeptidase N
FTW_2100	1702132-1703251	Major facilitator superfamily transport protein

[a]Annotation of corresponding allele in WY-00W4114 is denoted in parentheses.
