# Supplementary material for: Francisella tularensis Subtype A.II Genomic Plasticity in Comparison with Subtype A.I
Source: PLoS One. 2015 Apr 28;10(4):e0124906. doi: 10.1371/journal.pone.0124906 (PMC4412822; doi:10.1371/journal.pone.0124906)
Supplement: S10 Table — (PDF) [file pone.0124906.s011.pdf]

Additional file 11: Table S10. VNTR markers within the *F. tularensis* A.II genomes of WY-00W4114 and WY96-3416.

Sequence Motif (5'→3')	Nucleotide Repeat Size	WY-00W4114 Repeat Copy Number (Number of times in genome)	WY96-3418 Repeat Copy Number (Number of times in genome)
GT	2	5 (1)	5 (1)
		4 (6)	4 (6)
		3 (236)	3 (236)
AAT	3	4 (6)	4 (6)
		3 (220)	3 (219)
TTGTT	5	4 (1)	3 (1)
		2 (30)	2 (30)
TTTATA[b]	6	27 (1)	9 (1)
		2 (12)	2 (12)
TCATTA	6	3 (2)	3 (2)
		2 (5)	2 (5)
ATACTT	6	3 (1)	3 (1)
		2 (1)	2 (1)
CATTAA	6	2 (4)	2 (4)
AAAAAT	6	3 (1)	3 (1)
		2 (18)	2 (18)
TAAATA	6	27 (1)	9 (1)
		2 (13)	2 (13)
TATTTA	6	27 (1)	9 (1)
		2 (17)	2 (13)
TGATAAT[b]	7	2 (1)	3 (1)
TCAATTA	7	2 (2)	3 (1)
			2 (1)
AATATTTA[b]	8	3 (2)	2 (2)
AATAAGGAT	9	11(1)	2(1)
TAAAAGTAAG	10	1 (16)	1 (16)
TTTATATAAGT	11	2 (1)	2 (1)
ATTATTTTGATC	12	3 (1)	4 (1)
CTCCAGGACCAA[c]	12	1 (1)[c]	1 (1)[c]

TTATAAGTATTAA[b]	13	22 (1)	9 (1)
AAAGATATTTGTAGAA[a,b]	16	2 (37)	2 (37)
TTTCTACAAATATCTT	16	2 (37)	2 (37)
AAGAGCAAGAAAGTTCACCAA[b]	21	4 (1)	3 (1)
TTGGTGAACTTTCTTGCTCTT	21	4 (1)	3 (1)
AAGTAGCATTGTCACGACCTCCT[c]	23	1 (1)[c]	1 (1)[c]
AAGGGGAATTATCTAAACTTAGCCATTA[b]	28	2 (1)	1 (1)
AGATTCCAATCTGCGCAGTAATGACAGGTTTGG[b]	33	2 (1)	1 (1)
ATTGGTGAGCCTAGTTATGGTGAGGTTTTAGCAT ATCAAACACAAAGAAGAAGTATTGTT[b]	60	2 (1)	1 (48)
AGCTAAACCGCTGTTATTTAAGAGTTGAAAAGCA ATAAATATCAATGGTTTAGCAAATGA[b]	60	2 (1)	1 (9)

[a]Direct repeat flanking IS*Ftu1* ORFs.

[b]VNTR not present in the A.I genome.

[c]VNTR present in the A.I genome.
